# Colored tones of emotions: the relationship between music, emotions and color

**DOI:** 10.3389/fpsyg.2026.1777421

**Published:** 2026-09-16

**Authors:** Francesco Febbraio, Flavia De Simone, Cristina Taiani, Simona Collina

**Affiliations:** 1 Department of Education, Psychology and Communication, Suor Orsola Benincasa University, Naples, Italy; 2 Department of Classics, Sapienza University of Rome, Rome, Italy

**Keywords:** BPM, cross-modal Correspondence, emotions, HSL color space, music, Pleasure-Arousal-Dominance (PAD) 5, tonality

## Abstract

**Introduction:**

Music influences not only emotions but also cross-modal perception, including color. Previous studies have demonstrated that major keys and faster tempi are often associated with bright hues and positive affect, whereas minor keys and slower tempi tend to evoke darker shades and negatively valenced emotional states. However, how tempo and tonality jointly relate to emotional and chromatic responses has rarely been systematically examined, and the role of emotion in linking musical features to color choices remains underexplored.

**Methods:**

In this study, 80 participants with no formal musical training listened to 30-second excerpts derived from the 18th-century Neapolitan repertoire and other classical works. These excerpts varied in tonality (major/minor) and in the pitch class of their tonic (C-B), and each was presented at three tempi (-60, original, +60 BPM). Following each excerpt, participants reported an emotion in free-text form, which was mapped onto continuous Pleasure-Arousal-Dominance (PAD) scores using the NRC-VAD lexicon, and selected a color using an HSL-based picker; colors were analyzed in terms of Hue, Saturation and Lightness. Data were analyzed with generalized estimating equations accounting for repeated measures within participants.

**Results:**

Excerpts in major keys were associated with higher pleasure, whereas minor-key excerpts were associated with higher arousal, which increased at faster tempi; minor-key excerpts were also associated with darker color choices. Crucially, the emotional dimensions predicted color selection: pleasure was associated with more saturated and lighter colors, and arousal with darker colors. These associations remained stable after controlling for tonality, tempo, and absolute BPM.

**Discussion:**

These findings are consistent with an emotion-mediation account of music-color correspondences, whereby listeners form flexible, emotion-linked associations between sound and color.

## 1 Introduction

Music consists of sequences of notes that form melodic patterns assignable to major or minor tonalities. In music-theoretical terms, the two modes differ in the interval structure of their scales, most notably the third scale degree, which lies a major third (four semitones) above the tonic in major keys and a minor third (three semitones) in minor keys. These tonalities are often described as the quality capable of imparting an “emotional color” to a musical piece. A recent meta-analysis confirmed that this contrast remains a reliable predictor of listeners’ affective responses, although the strength of the association varies with factors such as age, musical training, and cultural background (Carraturo et al., 2025; Gagnon and Peretz, 2003; Hunter et al., 2010). Tempo, expressed in beats per minute (BPM), plays an equally central role: it regulates the speed of note succession and can influence emotional arousal, since faster tempi tend to energize or excite, whereas slower tempi are more likely to induce calmness or introspection (Iwanaga, 1995; Collier and Hubbard, 1998).

Beyond tonality and tempo, pitch represents another core quality of music capable of eliciting perceptual and affective responses during listening. Pitch refers to the perceptual attribute along which sounds are ordered from low to high frequency, primarily determined by the fundamental frequency of the tone. Pitch class, in turn, denotes the set of all pitches sharing the same note name across octaves (e.g., all Cs), and should be distinguished from tonality, which concerns the hierarchical organization of pitches around a tonal center rather than a property of individual tones. Cross-modal correspondences link pitch to visual and spatial attributes: tones of higher fundamental frequency are systematically associated with upper positions in space, and tones of lower frequency with lower positions (Eitan and Timmers, 2010). Such spatial mappings are complemented by associations with brightness, whereby higher pitches are consistently linked to brighter percepts (Eitan and Timmers, 2010; Eerola et al., 2012). From an emotional perspective, previous studies have shown that pitch height influences both pleasantness and arousal in listeners (Jaquet et al., 2014).

Differences among pitch classes (C, D, E, etc.) have also been shown to evoke coherent color associations in individuals with music-color synesthesia (Itoh et al., 2019). The use of a “musical note-color” Stroop test further demonstrated the automatic nature of these associations, as participants responded more slowly when the two dimensions were incongruent (Itoh et al., 2019). Neuroimaging studies have revealed that synaesthetes display enhanced activation during early auditory processing, a feature they share with individuals possessing absolute pitch (Loui et al., 2012). At later associative and visual processing stages, however, synaesthetes and absolute-pitch possessors differ from each other, suggesting that the neural pathways supporting sound-color mapping are only partially shared between the two groups (Loui et al., 2012). From a neurophysiological perspective, the latency of synaesthetic color experiences varies across individuals, emerging almost instantaneously in some and more gradually in others (Amsel et al., 2017). These findings highlight the need for flexible timing when studying cross-modal correspondences.

Pitch-color associations arise from both neurocognitive and cultural factors (Spence, 2020), and several mechanisms have been proposed to explain why attributes of sound and color become systematically linked. Spence (2011) distinguishes among structural correspondences, grounded in shared properties of neural coding; statistical correspondences, reflecting regularities of co-occurrence in the environment; and semantically mediated correspondences, based on shared linguistic labels applied to different modalities. A further route, particularly relevant to music, is affective mediation: auditory and visual stimuli may become associated because they elicit similar emotional states, so that music-color mappings are carried by the emotional qualities that the two stimuli share (Palmer et al., 2013; Malfatti et al., 2014; Lindborg and Friberg, 2015). The present study adopts this emotion-mediation perspective: rather than assuming direct mappings between acoustic and chromatic features, it examines whether the affective states evoked by music are systematically reflected in the properties of the colors listeners select.

Tonality, tempo (BPM), and the pitch-class quality of musical notes are not the only factors that shape immediate affective responses to music. Intrinsic characteristics of the listener, such as age and level of musical expertise, can also influence the ability to discriminate and recall melodies with greater accuracy compared to children or to those without formal musical education (Madsen and Madsen, 2002). However, this effect is not exclusive: younger listeners and individuals without musical training can still perceive and remember melodic structures reliably (Madsen and Madsen, 2002). Familiarity with a piece also matters. The more a melody is known, the stronger the emotional engagement tends to be (van den Bosch et al., 2013). Listening to familiar music has been shown to strengthen the connection between arousal and pleasure compared to unfamiliar pieces (van den Bosch et al., 2013). Considering these sources of variability, it is therefore essential to control for prior familiarity and musical expertise in studies on cross-modal associations by selecting unknown compositions and excluding participants with formal training when necessary. An often less explored but relevant aspect is the comparison between structured music and aleatoric sound, which functions as a kind of “amusical counterpart.” Aleatoric or indeterminate music, defined by intentional randomness and the suspension of conventional structure, challenges listeners’ expectations and alters affective engagement. Instead of predictable patterns that support aesthetic pleasure, such indeterminacy shifts perception toward more cognitively demanding and sometimes disruptive experiences (Huron, 2008; Wilson, 2023). A 2025 study suggests that musical training also affects the difference in perception between aleatoric and non-aleatoric musical fragments (Ponce-de-León et al., 2025), thereby emphasizing the importance of considering musical training as an exclusion criterion in cross-modal association studies that employ an “amusical” counterpart in their experiments.

There are several methods for evaluating emotions in response to music; among these, the use of free verbal labels has shown that they provide a better explanation than basic emotion and dimensional models (Zentner et al., 2008). Open-ended verbal reports or in-depth interviews have been used to assess the “strongest and most intense” experiences lived through music (Juslin and Sloboda, 2010), revealing a wide range of emotional, physical, cognitive, and spiritual reactions that extend beyond basic emotional categories (Juslin and Sloboda, 2010). Intercultural research has also used verbal labels to explore whether different cultures perceive similar emotions in unfamiliar music, and some emotional terms (e.g., sadness, joy, and fear) consistently emerge across groups (Balkwill and Thompson, 1999; Fritz et al., 2009). This approach makes it possible to evaluate emotional expressivity in response to music in a way that is spontaneous and adherent to the listener’s inner experience. Beyond these subjective and qualitative methods, research in Music Emotion Recognition (MER) has increasingly moved toward quantitative and standardized procedures, developing large-scale datasets to model emotional responses objectively (Aljanaki et al., 2017). Among these, the DEAM dataset (Database for Emotional Analysis of Music) is one of the most widely used benchmarks: it includes over 1,800 songs annotated according to the continuous dimensions of valence and arousal (Aljanaki et al., 2017), showing how these dimensions provide a valid alternative for assessing the emotional impact of music. Building on this dimensional perspective, the Pleasure–Arousal–Dominance (PAD) model developed by Russell and Mehrabian (1974) on the basis of semantic differential theory (Osgood et al., 1957) offers a comprehensive framework for describing affective states. The PAD framework identifies three core dimensions—Pleasure, Arousal, and Dominance—which together explain much of the variance in self-reported affect (Russell and Mehrabian, 1977; Shaver et al., 1987). Pleasure (or valence) captures the hedonic quality of an affective state, from unpleasant to pleasant; Arousal captures its degree of activation, from calm to excited; and Dominance captures the sense of control associated with the state, ranging from feeling controlled or overwhelmed by the situation to feeling in control of it. Together, these dimensions, allow emotions to be grouped into distinct profiles (e.g., high pleasure–low arousal–high dominance for comfort and relaxation, or low pleasure–high arousal–low dominance for hostility and anxiety). The PAD model was adopted in the present study for two reasons: first, the Dominance dimension distinguishes emotional states that valence and arousal alone cannot separate (e.g., anger and fear are both unpleasant and highly arousing, but differ markedly in perceived control); second, normative lexical resources provide PAD coordinates for freely produced verbal labels, allowing spontaneous emotion reports to be quantified within a common dimensional space (Mohammad, 2018). The PAD model therefore provides a structured foundation for investigating how musical features shape both emotional experience and cross-modal associations with color.

The literature recognizes that colors are not merely perceptual stimuli but carry systematic emotional associations. Red, frequently linked to arousal, intensity, and dominance (Elliot and Maier, 2014), is generally less pleasant than green and blue (Valdez and Mehrabian, 1994) and often evaluated with negative valence in performance and competitive contexts (Jonauskaite et al., 2024). By contrast, blue and green are more consistently associated with calmness and stability (Valdez and Mehrabian, 1994; Wilms and Oberfeld, 2018), and strongly connected to positive emotions and low-power states (Jonauskaite et al., 2024). Furthermore, blue has repeatedly been identified as the least activating color, closely tied to calmness and rest (Valdez and Mehrabian, 1994; Jonauskaite et al., 2024). Beyond single hues, cross-cultural studies have shown that the perceptual link between music, emotion, and color is both solid and specific. Major keys and faster tempi are commonly associated with brighter and more saturated colors, whereas minor keys and slower tempi relate to darker or more muted tones (Palmer et al., 2013). Tempo and tonality can therefore jointly modulate both emotional responses and color choices (Küssner et al., 2014). The same pattern emerges in the opposite direction, where bright and saturated colors are linked to positive emotions, unlike dark and desaturated tones associated with negative or low-arousal states (Valdez and Mehrabian, 1994). Meta-analyses confirm correspondences between specific hues and affective meanings across more than a century of research (Jonauskaite and Mohr, 2025). Taken together, these findings indicate with sufficient empirical robustness, that emotions elicited by music can influence color choices, configuring the music-emotion-color system as an interconnected set of cross-modal correspondences (Palmer et al., 2013). Recent studies have also extended this approach to generative art, systematically testing emotion-color mappings.

Although many studies have explored the links between music and emotion, and between music and color, it is still not clear how tonality and tempo act together or separately in relation to emotional responses and color choices. This study was designed to fill that gap by examining how these structural features are associated with participants’ self-reported emotions and color selections, analyzed both in terms of psychologically interpretable properties of the HSL color space (hue, saturation, and lightness) and, complementarily, as digital RGB channel values. To avoid confounding factors, all musical material was unfamiliar to participants, and individuals with formal musical training were not included. This approach was intended to limit the influence of familiarity and musical expertise on the observed associations. Participants provided free-text emotion terms, subsequently mapped onto continuous Pleasure, Arousal, and Dominance (PAD) scores through a normative affective lexicon for analysis. In parallel, they freely selected colors via a digital picker; these selections were decomposed into HSL properties and RGB channel values. This design tested whether core musical features are systematically associated with coordinated variations across affective and chromatic domains.

We formulated the following hypotheses:

*H1 (Emotions)*: Free-text emotion reports, quantified as continuous PAD scores, would differ across tempo and tonality conditions, with expected main effects and potential interactions.

*H2 (Colors)*: The chromatic properties of freely selected colors — saturation, lightness, and hue — together with the complementary RGB channel values, would likewise differ across tempo and tonality conditions, consistent with cross-modal correspondences between musical structure and color.

In addition, in line with the emotion-mediation account, we expected the affective dimensions themselves to be systematically associated with chromatic properties, in particular higher Pleasure with more saturated and lighter colors, and higher Arousal with darker colors (Valdez and Mehrabian, 1994; Palmer et al., 2013). Because each pitch class was represented by a single musical excerpt, differences across pitch classes were examined only as exploratory, excerpt-specific effects rather than as a formal hypothesis.

## 2 Materials and methods

### 2.1 Participants

Eighty native Italian speakers took part in the study, recruited predominantly among university students. No socio-demographic information (e.g., age, sex, education level, cultural background) was collected, as such data were not gathered at the time of testing; we explicitly acknowledge this as a limitation of the present study (see Section 4.8), and all findings should be interpreted as pertaining to the specific sample tested rather than to the general population. All participants reported normal or corrected-to-normal vision and hearing. During recruitment, participants were explicitly screened via self-report for auditory impairments and color-vision deficiencies (e.g., red-green deficiency), which constituted exclusion criteria; none of the enrolled participants reported such conditions. Because color-vision deficiencies were assessed through self-report only, undiagnosed deficiencies cannot be fully ruled out (see Section 4.8). Additional inclusion criteria required the absence of formal musical training and that participants were not regular listeners of classical music, so that the final sample consisted exclusively of non-expert listeners, the intended population of interest. In a debriefing at the end of the session, no participant reported recognizing the musical excerpts. Participation was voluntary, without monetary compensation or academic credit. The study was conducted in accordance with the Declaration of Helsinki. The research protocol was reviewed and approved by the Ethics Committee of Suor Orsola Benincasa University (protocol no. 8267), and written informed consent was obtained from all participants prior to the experimental procedure.

### 2.2 Stimuli and materials

To minimize potential familiarity effects, musical excerpts (Supplementary Table S1) were selected from the 18th-19th century Neapolitan classical repertoire (e.g., *N. Porpora, D. Scarlatti, Pergolesi*) as well as from other classical works that were not included in the concert programs of major international theaters such as *Teatro alla Scala, the Berliner Philharmoniker,* and the *New York Philharmonic*. Each excerpt was selected on the basis of the key of the piece: for each of the seven diatonic pitch classes (C, D, E, F, G, A, B), one piece written in the corresponding major key and one piece written in the corresponding minor key were selected (e.g., a piece in C major and a piece in C minor for pitch class C). The pitch-class factor therefore refers to the tonic of each excerpt’s key, not to isolated tones. Each pitch class was therefore represented by the same number of excerpts in each tonality and at each tempo, so that pitch class was balanced by design across all conditions. Importantly, because each pitch-class × tonality combination was represented by a single, distinct musical piece, effects associated with tonality and pitch class cannot be separated from other excerpt-level musical features (e.g., melody, instrumentation, register, rhythmic profile); all such effects are accordingly interpreted as excerpt-related associations rather than as intrinsic effects of tonality or pitch class (see Section 4.8). For every piece, three tempo conditions were created (original, −60 BPM, +60 BPM), resulting in a total of 42 excerpts. Tempo changes were implemented using Steinberg Cubase 12 Pro (Steinberg Media Technologies, 2022), with pitch-preserving time-stretching to ensure stable spectral properties across conditions. All excerpts were standardized to 30 s, a duration comparable to that used in previous work showing that affective evaluations of music are stable across excerpt durations (Bigand et al., 2005) and are formed rapidly after onset (Peretz et al., 1998); this duration also kept the overall session short enough to limit fatigue across trials. The original tempi of the stimuli were constrained between 80 and 120 BPM, an empirically supported range corresponding to spontaneous motor tempo and preferred musical tempi (Hine et al., 2022). This core range was extended by ±60 BPM to sample slower and faster conditions without reaching musically unrealistic extremes. As a result, absolute BPM values did not overlap across tempo categories (slow: 23–60 BPM; original: 83–120 BPM; fast: 143–180 BPM), although within-category variability was substantial; control analyses modeling tempo as a continuous absolute BPM value are reported in Section 3.6. Randomized filler segments from John *Cage’s Living Room Music* (Supplementary Table S1) were included after each trial. These aleatoric excerpts, characterized by the absence of stable harmonic structure and tempo regularity, served as an amusical contrast to reduce the predictability of the listening sequence, and were not included in the inferential analyses. Filler segments also lasted 30 s.

### 2.3 Hardware and software

Stimuli were presented on an iMac Pro (2017 model, 8-core Intel Xeon W processor, 32 GB RAM, 27-inch Retina 5K display, 5,120 x 2,880). Color selection was implemented through a custom HTML interface based on the HSL color space, consisting of a hue-saturation plane and a vertical lightness slider, rendered in the sRGB color space; selected colors were automatically recorded in hexadecimal format. The display was set to full brightness (100%) and ambient lighting was kept constant across participants (approximately 350 lux, 4,000 K). No instrument-based colorimetric calibration of the display was performed; this is acknowledged as a limitation (Section 4.8), although the use of a single device under constant viewing conditions ensured that color rendering was identical for all participants. Musical excerpts were delivered through Bose QuietComfort 45 headphones with active noise cancelation at a fixed playback volume (approximately 75 dB SPL). Emotion labels were entered via a standard QWERTY keyboard.

### 2.4 Procedure

After providing written informed consent, participants were seated approximately 60 cm from the display. A training phase preceded the main task, organized in three steps: participants first read the task instructions (reported in full in the Supplementary Appendix S1), then watched a short demonstration video illustrating the interface and the color-selection procedure, and finally completed three practice trials with non-experimental excerpts (Supplementary Table S1), providing both responses to familiarize themselves with the task. Once training was completed, the experiment began with the “start experiment” button. In the main task, participants listened to 30-s excerpts and provided two responses: (i) a single free-text emotion label in Italian, describing the affective state subjectively evoked by the music, typed via keyboard; and (ii) a color, freely selected through the HSL picker. Responses could be given at any moment during the excerpt, allowing both immediate and more reflective evaluations to be captured. At the end of each excerpt, a “Next” button appeared only after both responses had been entered, ensuring complete data for every trial. Task order (color first vs. emotion first) was counterbalanced across participants. The full session lasted approximately 28–30 min; no participant interrupted the session or requested breaks.

### 2.5 Experimental design

The study adopted a mixed factorial design with one between-subject factor (Tonality: major vs. minor) and two within-subject factors (Tempo: −60, original, +60 BPM; Pitch Class: C–B, operationalized as the key of the excerpt). Each participant completed 21 experimental trials (7 notes × 3 tempi) within the assigned tonality. Two families of dependent variables were considered: (i) emotional responses, obtained as free-text labels and quantified as Pleasure, Arousal and Dominance scores (Section 2.6.2); and (ii) chromatic responses, recorded as hexadecimal codes and analyzed both as RGB channel values and as HSL properties (Section 2.6.1). Aleatoric filler excerpts were excluded from inferential analyses.

### 2.6 Data analysis

Data preparation and analyses were conducted in R (version 4.5.2; R Core Team, 2025) using the packages geepack (Halekoh et al., 2006) and emmeans (Lenth, 2023); figures were produced with ggplot2 (Wickham, 2016). Given the repeated-measures structure of the data (21 observations per participant), all inferential models were estimated using Generalized Estimating Equations (GEE), specifying the participant identifier as the clustering unit, an exchangeable working correlation structure, and robust (sandwich) standard errors. Binomial models with a logit link were used for dichotomous outcomes (chromatic vs. achromatic responses), and Gaussian models with an identity link for continuous outcomes (RGB channels, Saturation, Lightness, circular Hue components, and PAD scores). Estimated marginal means with Bonferroni-adjusted pairwise comparisons were used to follow up significant effects. Because GEE inference relies on robust (sandwich) standard errors and is asymptotic, all estimated marginal means and pairwise contrasts are reported with z statistics rather than with residual-based t statistics. The robustness of all main models was assessed by re-estimating them under an independence working correlation structure, and additional control analyses modeled tempo as a continuous absolute BPM value and included task order (color first vs. emotion first) as a covariate. Because each design cell contained a single musical excerpt, excerpt-level random variability could not be estimated separately from condition effects; pitch-class effects were therefore treated as exploratory, excerpt-specific effects. The affective dimensions and the HSL color properties were treated as primary outcomes; RGB channels were complementary (Section 3.5), and analyses of pitch class and individual emotion labels were exploratory. Pairwise comparisons were Bonferroni-adjusted within each family of contrasts; no correction was applied across families, as these outcomes are not mutually independent and the complementary and exploratory analyses are interpreted descriptively. All tests were two-tailed with *α* = 0.05.

#### 2.6.1 Color data

Chromatic responses were recorded in hexadecimal format as the direct output of the digital picker and were converted to RGB values (0–255), the fundamental channels of the sRGB digital color space (Fairchild, 2013). From the RGB values, responses were further transformed into the HSL space, which decomposes color into psychologically interpretable properties: Hue (chromatic tone, expressed in degrees on a circular 0–360° scale), Saturation (chromatic intensity, 0–100), and Lightness (perceived luminosity, 0–100) (Palmer, 1999; Fairchild, 2013). The RGB → HSL conversion was performed through standard deterministic formulas (Fairchild, 2013). Because Hue is a circular variable, it was not treated as a linear predictor or outcome. Following established methodological practice for circular data (Fisher, 1995; Berens, 2009), Hue was decomposed into its sine and cosine components (Hue_sin, Hue_cos) for inferential modeling, and circular descriptive statistics (mean direction θ̄ and mean resultant length R) were computed. Prior to the Hue analyses, responses were classified as chromatic or achromatic: selections with very low saturation (Saturation < 5) were considered achromatic (grays, whites, blacks) and analyzed separately, as their hue is not meaningfully defined, consistent with the practice of treating achromatic responses separately in color-emotion research (Wilms and Oberfeld, 2018). The value of 5 on the 0–100 saturation scale was adopted as a conservative operational threshold: below it, selections are visually indistinguishable from gray and the hue angle becomes numerically unstable, since hue is undefined at zero saturation. Three levels of the chromatic response were thus analyzed: (i) the probability of producing a chromatic rather than an achromatic response; (ii) the continuous properties Saturation and Lightness; and (iii) the chromatic orientation of the selected hues, modeled through the circular components. RGB channel values were additionally analyzed as a complementary level of description, treating them not as perceptual measures but as behavioral outputs of participants’ color choices at the level of the digital output channels. Because RGB channels are device-oriented, interdependent quantities that do not directly index perceptual color dimensions, the HSL analyses constitute the primary chromatic outcomes of the study.

#### 2.6.2 Emotion data

Participants’ responses consisted of free verbal labels in Italian. Labels first underwent conservative formal normalization (removal of extra spaces and capitalization; unification of clearly equivalent inflected forms, e.g., “ansiogeno,” “in ansia,” “ANSIA” → “ansia”), without any interpretive merging of distinct concepts. Across the 1,680 responses, participants produced 164 distinct normalized Italian labels. Each label was then translated into English using the Oxford English Dictionary through direct lexical correspondence, yielding 159 distinct English terms (the complete mapping is reported in Supplementary Table S2), and matched against the NRC Valence–Arousal–Dominance Lexicon, version 2.1 (Mohammad, 2018, 2025), a normative lexicon providing human-annotated Valence, Arousal, and Dominance ratings for approximately 55,000 English terms, collected via Best-Worst Scaling. The extended coverage of version 2.1 ensured an exact lexicon match for all translated labels, with no missing values. Lexicon scores, natively expressed on a −1 to +1 scale, were linearly rescaled to 0–1. Each trial thus contributed a triplet of continuous Pleasure, Arousal, and Dominance scores, which served as the affective dependent variables in all analyses; no binary recoding or arbitrary thresholds were applied. In addition, emotion labels were independently coded by the first author into three binary PAD indicators (presence vs. absence of each dimension) using a rule-based scheme. This manual coding served as a robustness check: convergent-validity analyses showed very high agreement between the manual coding and the NRC-VAD scores (point-biserial correlations: Pleasure *r* = 0.951, Arousal *r* = 0.863, Dominance *r* = 0.654; all *p* < 0.001, *N* = 1,680; Cohen’s *d* for the separation between presence/absence codes: 6.12, 3.42, and 1.73, respectively; word-level correlations: *r* = 0.855, 0.723, 0.634, *N* = 159). In addition, a second independent rater, naive to the aims of the study, coded all 164 distinct Italian labels using a written codebook (Supplementary Appendix S2). Inter-rater agreement with the first author’s coding (aggregated at the word level by majority) was excellent for Pleasure (*κ* = 0.85, 95% CI [0.77, 0.93]; agreement = 93%), moderate for Dominance (*κ* = 0.59 [0.46, 0.71]; agreement = 79%), and low for Arousal (*κ* = 0.28 [0.11, 0.44]; agreement = 68%; prevalence-adjusted bias-adjusted kappa, PABAK = 0.37). The two raters differed in how broadly they applied the activation criterion (80% vs. 60% positive codes), and their codings related differently to the NRC-VAD norms (word-level *r* = 0.35 and 0.86, respectively). The discrepancy between the low kappa and the substantial raw agreement for Arousal reflects the well-known sensitivity of kappa to unbalanced marginal prevalence (Byrt et al., 1993): because both coders classified most words as arousing, the prevalence-adjusted PABAK provides a less deflated estimate of agreement. The binary classification of arousal therefore appears sensitive to individual response criteria, and its reliability remains limited. Because the analyses reported below rely on the continuous NRC-VAD scores rather than on the binary coding, this limitation does not affect the primary results, but it should be borne in mind when interpreting the convergent-validity analyses involving Arousal.

## 3 Results

### 3.1 Descriptive statistics

Across the 1,680 experimental trials, participants produced 164 distinct normalized Italian emotion labels (159 distinct English lexicon entries). Mean NRC-VAD scores were *M* = 0.52 (SD = 0.38) for Pleasure, *M* = 0.51 (SD = 0.31) for Arousal, and *M* = 0.46 (SD = 0.22) for Dominance (scale 0–1). Most color responses were chromatic rather than achromatic: 89.8% of selections (1,509 of 1,680) exceeded the saturation threshold (*S* ≥ 5). Mean RGB values were 145.68 (SD = 93.52), 137.23 (SD = 92.93), and 140.90 (SD = 92.91) for the red, green, and blue channels, respectively. Among chromatic responses, colors were on average highly saturated (Saturation: *M* = 89.15, SD = 22.17) and of intermediate lightness (Lightness: *M* = 58.98, SD = 18.66). Table 1 report estimated marginal means and the corresponding pairwise comparisions by condition.

**Table 1:** Estimated marginal means (95% CI) for the PAD dimensions by tonality and tempo condition.

Tonality	Tempo	Pleasure	Arousal	Dominance
Major	−60 BPM	0.538 [0.478, 0.597]	0.398 [0.360, 0.435]	0.421 [0.393, 0.449]
Major	Original	0.663 [0.618, 0.708]	0.455 [0.414, 0.497]	0.503 [0.475, 0.530]
Major	+60 BPM	0.580 [0.526, 0.634]	0.466 [0.422, 0.509]	0.468 [0.436, 0.500]
Minor	−60 BPM	0.338 [0.290, 0.386]	0.528 [0.492, 0.563]	0.388 [0.363, 0.413]
Minor	Original	0.498 [0.452, 0.544]	0.577 [0.535, 0.619]	0.481 [0.455, 0.506]
Minor	+60 BPM	0.497 [0.443, 0.551]	0.655 [0.619, 0.690]	0.507 [0.477, 0.538]

Estimated marginal means from Gaussian GEE models (Tonality × Tempo) with participants as clustering units and robust standard errors. PAD scores are continuous NRC-VAD values (0–1). Brackets contain 95% confidence intervals.

### 3.2 Emotional responses: effects of tonality and tempo on PAD dimensions

Continuous Pleasure, Arousal, and Dominance scores were modeled with Gaussian GEEs including Tonality, Tempo, and their interaction; estimated marginal means (EMMs) with Bonferroni-adjusted pairwise comparisons are shown in Figure 1 and Table 2.

**Figure 1:**
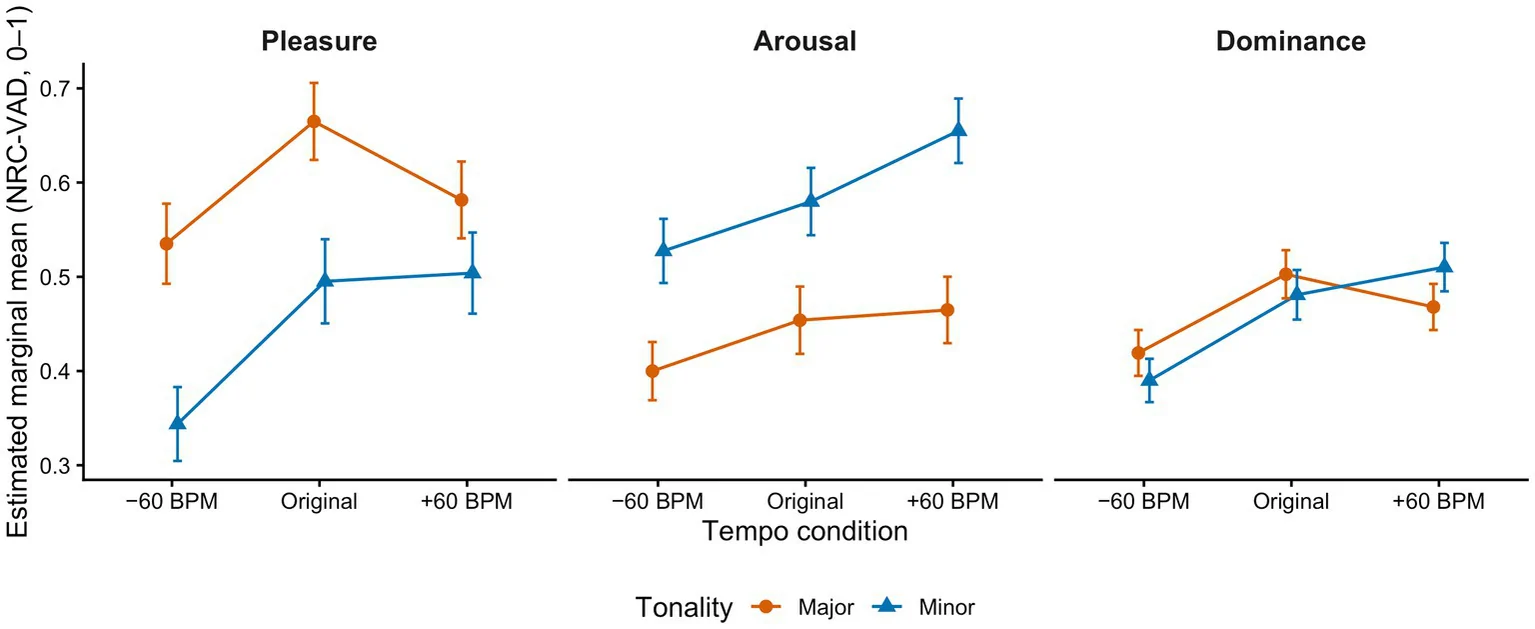
Estimated marginal means of pleasure, arousal, and dominance (NRC-VAD scores, 0–1) by tonality and tempo condition. Error bars represent 95% confidence intervals from GEE models with participants as clustering units.

**Table 2:** Bonferroni-adjusted pairwise comparisons for the PAD dimensions by tempo and tonality.

Dimension	Tonality	Comparison	Estimate	SE	*z*	*p*
Pleasure						
	Major	−60 vs. Original	−0.125	0.032	−3.89	<0.001
	Major	−60 vs. +60	−0.042	0.019	−2.28	0.068
	Major	Original vs. +60	0.083	0.033	2.51	0.036
	Minor	−60 vs. Original	−0.160	0.022	−7.33	<0.001
	Minor	−60 vs. +60	−0.159	0.029	−5.56	<0.001
	Minor	Original vs. +60	0.001	0.024	0.05	1.000
Arousal						
	Major	−60 vs. Original	−0.058	0.018	−3.16	0.005
	Major	−60 vs. +60	−0.068	0.020	−3.43	0.002
	Major	Original vs. +60	−0.011	0.016	−0.67	1.000
	Minor	−60 vs. Original	−0.049	0.022	−2.25	0.074
	Minor	−60 vs. +60	−0.127	0.012	−10.24	<0.001
	Minor	Original vs. +60	−0.078	0.021	−3.76	<0.001
Dominance						
	Major	−60 vs. Original	−0.082	0.017	−4.80	<0.001
	Major	−60 vs. +60	−0.047	0.013	−3.69	<0.001
	Major	Original vs. +60	0.035	0.019	1.83	0.205
	Minor	−60 vs. Original	−0.093	0.015	−6.35	<0.001
	Minor	−60 vs. +60	−0.119	0.017	−6.83	<0.001
	Minor	Original vs. +60	−0.027	0.013	−1.98	0.144
Major vs. Minor (by tempo)						
Pleasure		−60/Original/+60	0.199/0.165/0.083	0.039/0.033/0.039	5.13/5.03/2.14	<0.001/<0.001/0.033
Arousal		−60/Original/+60	−0.130/−0.122/−0.189	0.026/0.030/0.029	−4.96/−4.06/−6.56	<0.001/<0.001/<0.001
Dominance		−60/Original/+60	0.033/0.022/−0.039	0.019/0.019/0.023	1.73/1.14/−1.75	0.085/0.254/0.081

Contrasts on estimated marginal means from Gaussian GEE models; *p*-values Bonferroni-adjusted within each family of comparisons. Because GEE inference is asymptotic and based on robust standard errors, contrasts are tested with *z* statistics. Estimates on the NRC-VAD 0–1 scale.

#### 3.2.1 Pleasure

Pleasure was higher for major-key than minor-key excerpts (Tonality: *β* = −0.199, SE = 0.039, Wald = 26.28, *p* < 0.001), at every tempo condition (−60 BPM: Δ = 0.199, *p* < 0.001; original: Δ = 0.165, *p* < 0.001; +60 BPM: Δ = 0.083, *p* = 0.033). The association between tempo and Pleasure differed across tonalities (Tonality × Tempo at +60 BPM: *β* = 0.116, SE = 0.034, Wald = 11.67, *p* < 0.001). In the major key, Pleasure peaked at the original tempo (EMMs: 0.538, 0.663, 0.580 for −60, original, and +60 BPM), which was higher than both the slowed (*p* < 0.001) and the accelerated condition (*p* = 0.037). In the minor key, Pleasure increased from the slowed condition (EMM = 0.338) to the original tempo (0.498, *p* < 0.001) and remained at that level at +60 BPM (0.497; original vs. +60: *p* = 1.00).

#### 3.2.2 Arousal

Arousal was higher for minor-key than major-key excerpts at every tempo (all *p* < 0.001; e.g., +60 BPM: EMM = 0.655 vs. 0.466). In the minor key, Arousal increased with tempo, reaching its maximum in the accelerated condition (−60 vs. +60 BPM: Δ = 0.127, *p* < 0.001; original vs. +60: Δ = 0.078, *p* < 0.001), whereas in the major key it increased from the slowed to the original tempo (*p* = 0.005) with no further increase at +60 BPM (*p* = 1.00). The Tonality × Tempo interaction at +60 BPM was significant (*β* = 0.059, SE = 0.023, Wald = 6.24, *p* = 0.012).

#### 3.2.3 Dominance

Dominance increased from the slowed to the original tempo in both tonalities (major: Δ = 0.082, *p* < 0.001; minor: Δ = 0.093, *p* < 0.001), with no further significant change at +60 BPM (*p* = 0.205 and *p* = 0.144, respectively). Notably, major and minor keys did not differ significantly in Dominance at any tempo (all *p* ≥ 0.081).

### 3.3 Chromatic responses

#### 3.3.1 Chromatic versus achromatic selections

A binomial GEE on the probability of producing a chromatic response revealed significant effects of both tonality and tempo. Chromatic selections were less likely for minor-key excerpts (*β* = −0.904, SE = 0.337, Wald = 7.21, *p* = 0.007) and more likely at faster tempi relative to the slowed condition (original: *β* = 0.902, SE = 0.289, Wald = 9.73, *p* = 0.002; +60 BPM: *β* = 1.406, SE = 0.303, Wald = 21.60, *p* < 0.001). Tonality × Tempo interactions were not significant (all *p* ≥ 0.26), indicating that the two factors operated additively. Estimated marginal probabilities of a chromatic response were 0.89 [0.83, 0.94] at the slowed, 0.95 [0.91, 0.98] at the original, and 0.97 [0.94, 0.99] at the accelerated tempo for major-key excerpts, and 0.77 [0.70, 0.83], 0.90 [0.84, 0.94] and 0.90 [0.84, 0.94] respectively for minor-key excerpts. Within each tonality, chromatic responses were less likely at the slowed than at the original tempo (major: OR = 0.41, *p* = 0.005; minor: OR = 0.38, *p* < 0.001) and then at the accelerated tempo (major: OR = 0.25, *p* < 0.001; minor: OR = 0.38, *p* < 0.001), with no difference between the original and accelerated conditions (both *p* ≥ 0.37).

#### 3.3.2 Saturation and lightness

Among chromatic responses, saturation values were distributed across the full scale but markedly concentrated toward high values, whereas lightness clustered in the intermediate range (approximately *L* = 50–60), with participants generally avoiding both very dark and near-white selections (Figures 2, 3). Saturation and Lightness were then modeled as a function of tonality and tempo. Saturation increased with tempo in the minor key (slowed vs. accelerated: Δ = 7.53, *p* = 0.005), with no reliable tempo differences in the major key (all *p* ≥ 0.07); major-key excerpts were associated with more saturated colors at the original tempo (Δ = 5.39, *p* = 0.027) but not at the slowed or accelerated tempo. Lightness showed a clearer pattern: major-key excerpts were associated with lighter colors at every tempo (Δ = 11.07, 10.17 and 5.92 at the slowed, original and accelerated tempo, respectively; all *p* ≤ 0.005), replicating the association between minor mode and darker color choices reported by Palmer et al. (2013). Within the minor key, Lightness increased from the slowed to the original (Δ = 4.02, *p* = 0.046) and to the accelerated tempo (Δ = 4.61, *p* = 0.012), whereas in the major key it decreased from the original to the accelerated condition (Δ = 3.66, *p* = 0.026).

**Figure 2:**
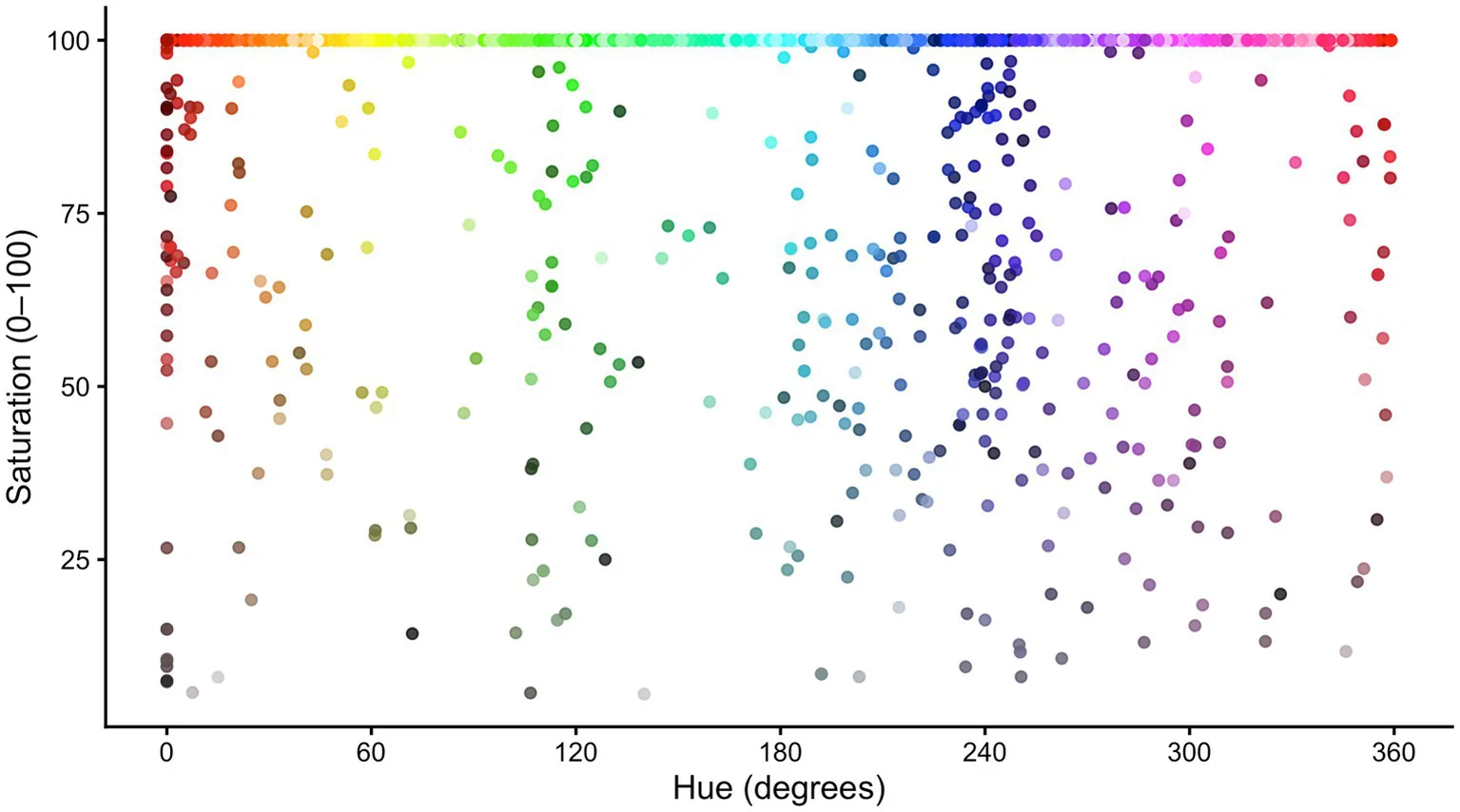
Distribution of chromatic responses in the Hue-Saturation plane. Each point represents one color selection, displayed in the actual color chosen by the participant. Chromatic responses only (*S* ≥ 5; *N* = 1,509).

**Figure 3:**
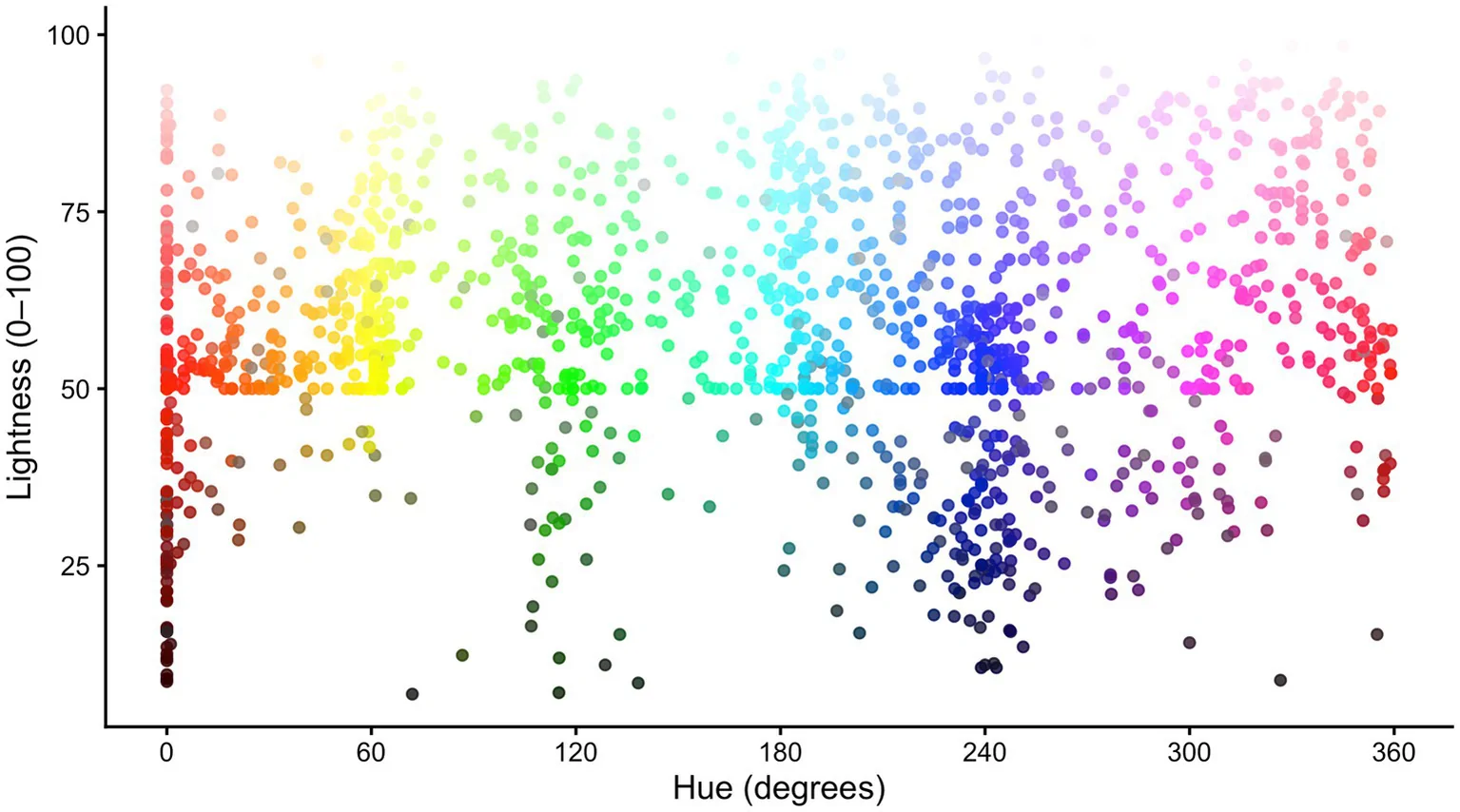
Distribution of chromatic responses in the Hue-Lightness plane. Each point represents one color selection, displayed in the actual color chosen by the participant. Chromatic responses only (*S* ≥ 5; *N* = 1,509).

#### 3.3.3 Global distributions of hue

The circular distribution of Hue was widely dispersed (mean direction θ̄ = 288°, mean resultant length *R* = 0.083; Figure 4). The very low angular concentration indicates that color selections did not converge on a globally dominant hue; the mean direction should therefore be read only as a synthetic descriptive index. This absence of a dominant hue at the aggregate level makes the relationship between hue and affective dimensions, examined below, particularly informative. Modeled as a function of tonality and tempo, the circular components showed more limited effects. In the minor key, faster tempi were associated with a shift toward the red region (cosine component, slowed vs. accelerated: Δ = 0.20, *p* = 0.002; original vs. accelerated: Δ = 0.14, *p* = 0.008), and at the accelerated tempo minor-key excerpts were oriented more strongly toward red than major-key excerpts (Δ = 0.20, *p* = 0.004). No reliable tonality differences emerged at the slowed or original tempo.

**Figure 4:**
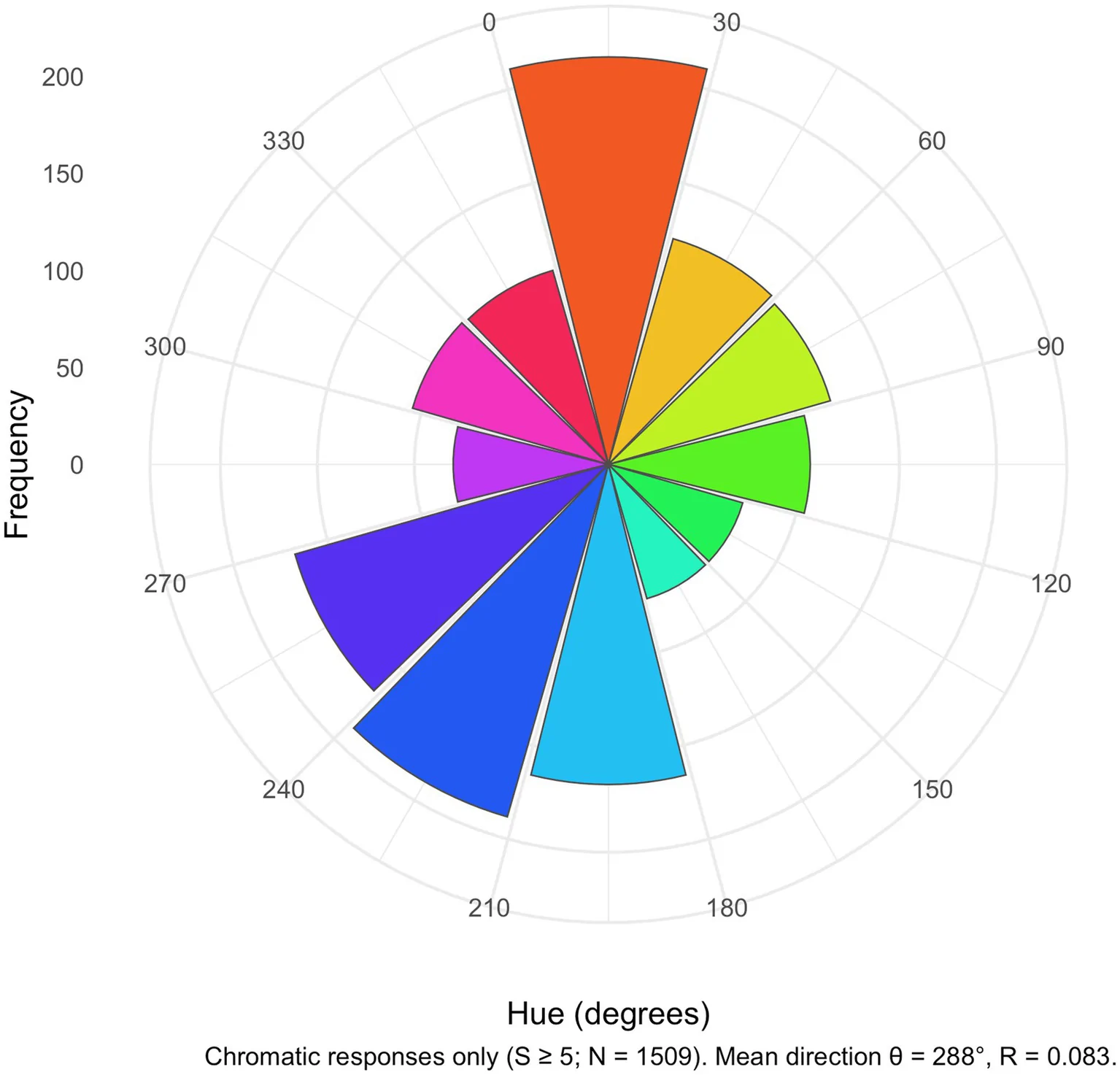
Circular distribution of Hue (12 bins of 30°) for chromatic responses (*S* ≥ 5; *N* = 1,509). Although the circular mean direction was *θ* = 288°, the angular concentration was very low (*R* = 0.083), indicating a widely dispersed distribution with no globally dominant hue.

### 3.4 Affect-color associations

#### 3.4.1 PAD dimensions and saturation/lightness

Gaussian GEEs on chromatic responses modeled Saturation and Lightness as a function of the three continuous PAD dimensions (Table 3 and Figure 5). Pleasure was the only dimension significantly associated with Saturation (*β* = 18.00, SE = 2.86, Wald = 39.58, *p* < 0.001; Arousal and Dominance: *p* ≥ 0.26): a + 0.10 increment in Pleasure corresponded to an expected increase of +1.80 Saturation points (95% CI [1.24, 2.36]). For Lightness, Pleasure showed a positive association (*β* = 18.90, SE = 2.93, Wald = 41.72, *p* < 0.001; +1.89 points per +0.10 increment, 95% CI [1.32, 2.46]), whereas Arousal was negatively associated (*β* = −7.64, SE = 2.49, Wald = 9.43, *p* = 0.002; −0.76 points per +0.10, 95% CI [−1.25, −0.28]); Dominance showed a negative but non-significant trend (*β* = −8.50, SE = 4.67, *p* = 0.068). Thus, more pleasant affective states were associated with more saturated and lighter colors, and more aroused states with darker colors. Sensitivity analyses adding Tonality and Tempo as covariates left these associations unchanged indicating that the affect-color relationships are not reducible to the musical effects reported in Section 3.3. Re-estimating all models (including the RGB models reported in Section 3.5) under an independence working correlation structure left the direction and significance of every reported effect unchanged. Point estimates were identical for the PAD models and differed by at most 2.6 points for the Saturation and Lightness models (0–100 scale) and 6.8 points for the RGB models (0–255 scale), that is, by less than 3% of the respective scales.

**Table 3:** GEE coefficients for the associations between PAD dimensions and chromatic properties.

Outcome	Predictor	*β*	SE	Wald	*p*
Saturation	Pleasure	18.00	2.86	39.58	<0.001
	Arousal	1.19	2.67	0.20	0.657
	Dominance	4.54	4.02	1.27	0.259
Lightness	Pleasure	18.90	2.93	41.72	<0.001
	Arousal	−7.64	2.49	9.43	0.002
	Dominance	−8.50	4.67	3.32	0.068
Hue_sin	Pleasure	0.51	0.10	28.54	<0.001
	Arousal	0.22	0.09	5.91	0.015
	Dominance	0.26	0.17	2.27	0.132
Hue_cos	Pleasure	0.00	0.12	0.00	0.977
	Arousal	0.44	0.11	15.85	<0.001
	Dominance	0.43	0.23	3.40	0.065
Red	Pleasure	64.57	15.14	18.19	<0.001
	Arousal	13.50	12.78	1.11	0.291
	Dominance	30.09	27.61	1.19	0.276
Green	Pleasure	121.68	12.62	92.91	<0.001
	Arousal	−23.69	13.62	3.02	0.082
	Dominance	−36.46	26.94	1.83	0.176
Blue	Pleasure	46.08	13.22	12.14	<0.001
	Arousal	−57.14	12.70	20.24	<0.001
	Dominance	−96.80	24.24	15.95	<0.001

Gaussian GEE models with participants as clustering units, exchangeable working correlation, and robust standard errors. Predictors are continuous NRC-VAD scores (0–1); each β expresses the expected change in the outcome for a full-scale (0 → 1) change in the predictor. Saturation, Lightness, and Hue models include chromatic responses only (*S* ≥ 5, *n* = 1,509); RGB models include all responses (*n* = 1,680). Pleasure and Dominance scores were correlated (*r* = 0.69); multivariate Dominance coefficients should be interpreted accordingly.

**Figure 5:**
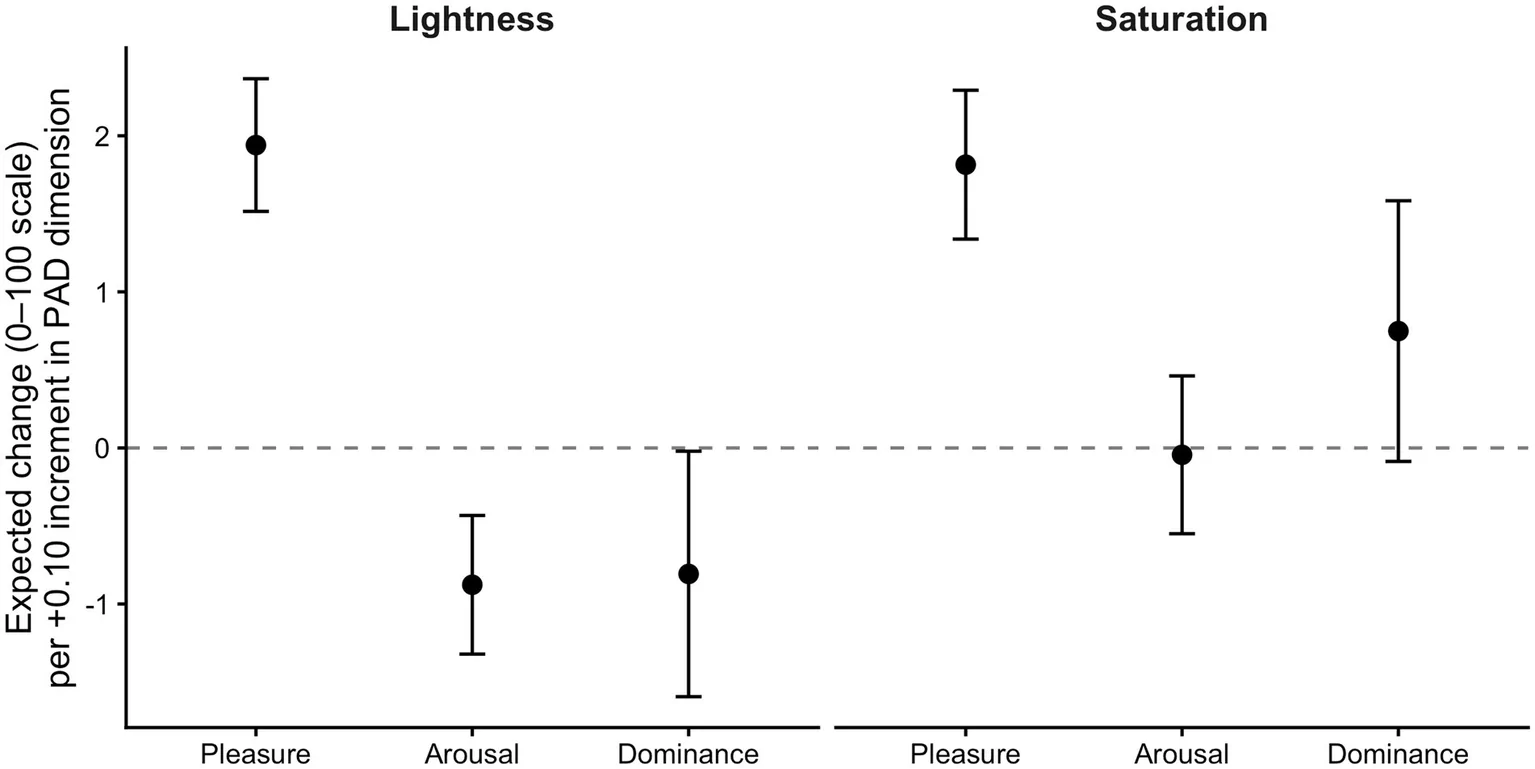
Expected change in saturation and lightness (0–100 scale) for a + 0.10 increment in each PAD dimension, estimated from GEE models on chromatic responses, holding the other dimensions constant. Error bars represent 95% confidence intervals.

#### 3.4.2 PAD dimensions and hue

Hue was analyzed through its circular components (Table 3). For the sine component, Pleasure showed a significant positive association (*β* = 0.511, SE = 0.096, Wald = 28.54, *p* < 0.001), as did Arousal to a smaller extent (*β* = 0.224, SE = 0.092, Wald = 5.91, *p* = 0.015); Dominance was not significant. For the cosine component, only Arousal was significant (*β* = 0.438, SE = 0.110, Wald = 15.85, *p* < 0.001; Dominance: *p* = 0.065). In terms of chromatic orientation, positive sine values correspond to a displacement toward the 90° region (yellow-green), and positive cosine values toward 0°/360° (red): higher Pleasure was thus associated with a shift toward warmer yellow-green hues, and higher Arousal with a shift toward the red sector. Given the highly dispersed global distribution (*R* = 0.083), these associations should be interpreted as relative directional tendencies within a heterogeneous chromatic space rather than as convergence on discrete hues.

#### 3.4.3 Exploratory analysis of frequent emotion labels

As an exploratory, descriptive analysis, we examined the 10 most frequent emotion labels, which together accounted for 862 of the 1,680 responses (51.3%), with between 56 and 151 responses per label (Table 4). These labels spanned almost the entire Pleasure range of the lexicon, from 0.05 (sadness) to 0.98 (joy), and this gradient was mirrored in the corresponding color choices: labels with high normative Pleasure, such as joy and cheerfulness, were associated almost exclusively with chromatic responses and with high mean Saturation and Lightness, whereas labels with low Pleasure, such as sadness and anxiety, showed a higher proportion of achromatic selections and lower mean Saturation and Lightness. Within-label circular statistics revealed that some labels showed marked hue convergence: anger was associated with the red-magenta sector (θ̄ ≈ 345°) and sadness with the blue-cyan sector (θ̄ ≈ 231°), both with comparatively high angular concentration (Figure 6). These label-level profiles indicate that, although no hue dominated globally, specific emotional meanings were reliably expressed through specific regions of color space. Because the labels showing the highest angular concentration were identified after inspecting the data, these analyses are descriptive and *post hoc*, and the corresponding hue-label associations require confirmation in an independent sample.

**Table 4:** Ten most frequent emotion labels, with normative PAD values and associated color properties.

Label	*n*	%	Pleasure	Arousal	Dominance	% chromatic	Saturation	Lightness
Sadness	151	9.0	0.052	0.288	0.164	74.2	77.4	53.6
Calm	132	7.9	0.875	0.050	0.314	95.5	95.3	68.0
Melancholy	93	5.5	0.188	0.260	0.237	94.6	78.7	54.2
Anxiety	88	5.2	0.146	0.865	0.342	84.1	79.9	45.5
Joy	85	5.1	0.980	0.824	0.794	100.0	98.0	61.3
Happiness	75	4.5	0.960	0.732	0.850	98.7	99.3	62.2
Tranquility	63	3.8	0.840	0.135	0.442	98.4	94.3	69.8
Cheerfulness	61	3.6	0.969	0.802	0.675	100.0	99.5	65.7
Serenity	58	3.5	0.900	0.061	0.557	98.3	93.2	66.2
Anger	56	3.3	0.167	0.865	0.657	82.1	90.0	46.3

n = number of responses; % = proportion of the 1,680 experimental trials. Pleasure, Arousal and Dominance are normative NRC-VAD values for the corresponding English term (0–1 scale) and are therefore constant within each label. % chromatic = proportion of responses to that label exceeding the saturation threshold (*S* ≥ 5); Saturation and Lightness are means computed over chromatic responses only. Labels are ordered by frequency.

**Figure 6:**
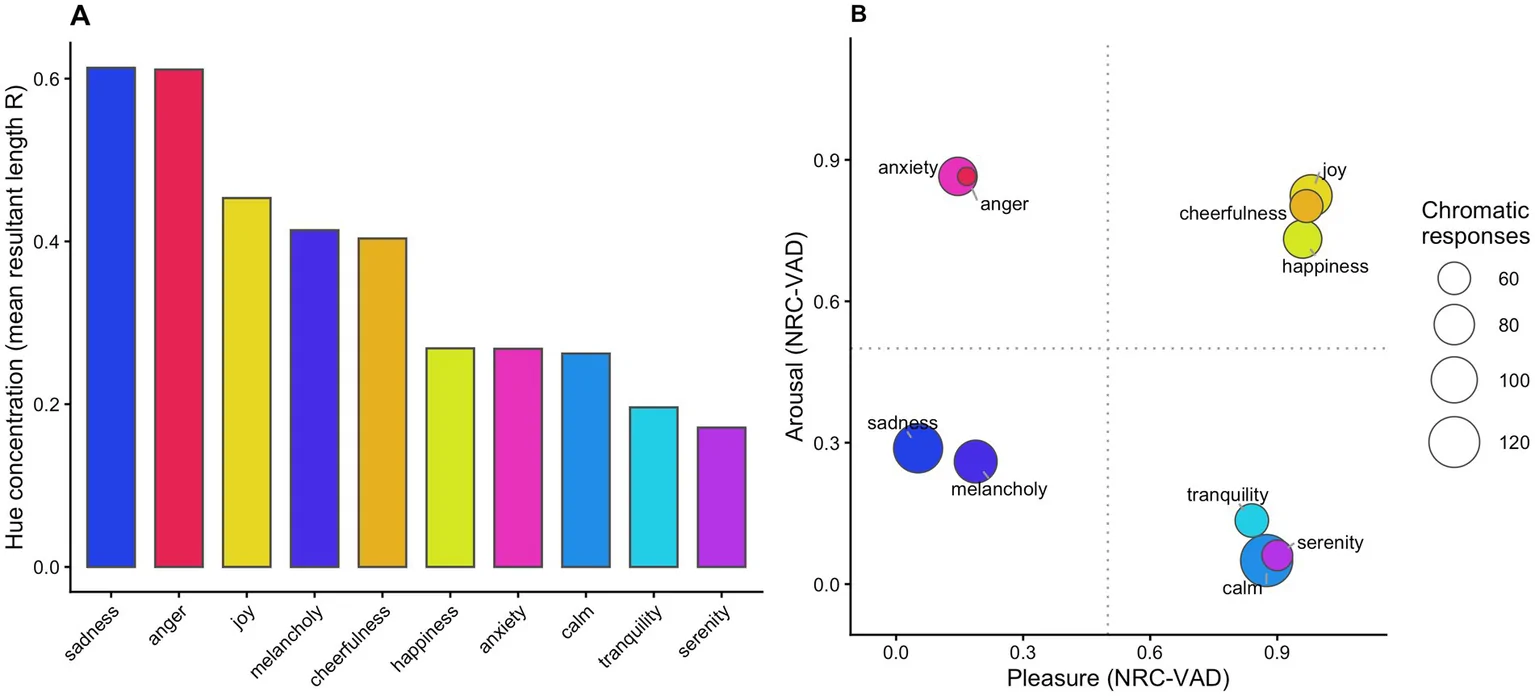
Ten most frequent emotion labels. **(A)** Hue concentration (mean resultant length R) for each label, computed over chromatic responses; bar color corresponds to the mean hue direction of that label. **(B)** Position of the same labels in the Pleasure × Arousal space defined by the normative NRC-VAD values; point size is proportional to the number of chromatic responses and point color corresponds to the mean hue direction. Dotted lines mark the scale midpoints.

### 3.5 Complementary analyses: RGB channels

Because participants’ color selections were recorded as RGB triplets, we also examined the three channels directly. RGB values are a deterministic transformation of the HSL coordinates and therefore add no independent information; they are reported to document the format in which responses were stored and to allow comparison with earlier studies using RGB. The affective pattern mirrored the HSL results (Table 3): Pleasure was positively associated with all three channels (Red: *β* = 64.57; Green: *β* = 121.68; Blue: *β* = 46.08; all *p* < 0.001), consistent with the positive Pleasure-Lightness association observed in HSL space. Arousal negatively with Blue (*β* = −57.14, *p* < 0.001) and, as a non-significant trend, with Green (*β* = −23.69, *p* = 0.082), and Dominance negatively with Blue (*β* = −96.80, *p* < 0.001), a pattern corresponding to warmer and darker selections at higher levels of activation and control. Given the correlation between Pleasure and Dominance (*r* = 0.69), the multivariate Dominance coefficients should be interpreted with caution. Tonality and tempo effects on the individual channels mirrored the corresponding HSL results and are not reported separately.

### 3.6 Control analyses: absolute tempo and task order

Because tempo conditions were defined relative to each excerpt’s original tempo, absolute BPM values varied within categories (slow: 23–60; original: 83–120; fast: 143–180 BPM), although the categories did not overlap. To verify that tempo effects were not an artifact of this categorization, all main models were re-estimated with tempo as a continuous absolute BPM predictor. All effects were confirmed: for each +10 BPM, Pleasure increased by 0.009 (*p* < 0.001), Arousal by 0.005 (*p* = 0.002), and Dominance by 0.006 (*p* < 0.001); the probability of a chromatic response increased (*β* = 0.107, *p* < 0.001; OR ≈ 1.11 per +10 BPM), as did Saturation (+0.29 points, *p* = 0.010) and Lightness (+0.17 points, *p* = 0.035). The associations between PAD dimensions and chromatic properties remained unchanged when absolute BPM was included as a covariate.

#### 3.6.1 Task order

Response order (color first vs. emotion first) did not predict any affective or chromatic outcome (all *p* ≥ 0.16), and including it as a covariate left the affect–color coefficients essentially unchanged (e.g., Pleasure on Lightness: β = 18.86 vs. 18.90). Order × PAD interactions were not reliable (all *p* ≥ 0.17), with the exception of a marginal Arousal × Order term for Lightness (*p* = 0.053).

### 3.7 Summary of results

Taken together, the results show that tonality and tempo were systematically associated with both affective and chromatic responses. Major-key excerpts were associated with higher Pleasure at every tempo and with a higher probability of chromatic selection; minor-key excerpts with higher Arousal and lower Lightness. Faster tempi were associated with higher Arousal (most consistently in the minor key), Dominance, the probability of chromatic responses, and Saturation, with all effects confirmed when tempo was modeled as absolute BPM. In the affective-chromatic mapping, Pleasure was associated with more saturated and lighter colors, Arousal with darker colors and a directional shift toward red hues, while label-level analyses showed reliable hue convergence for specific emotions (anger → red-magenta; sadness → blue-cyan). These findings support both hypotheses (H1 and H2), indicating systematic associations between musical structure, affective dimensions, and color choices, and are consistent with an emotion-mediated account of music-color correspondences. Figure 7 and Table 5 summarize the direction of the observed associations.

**Figure 7:**
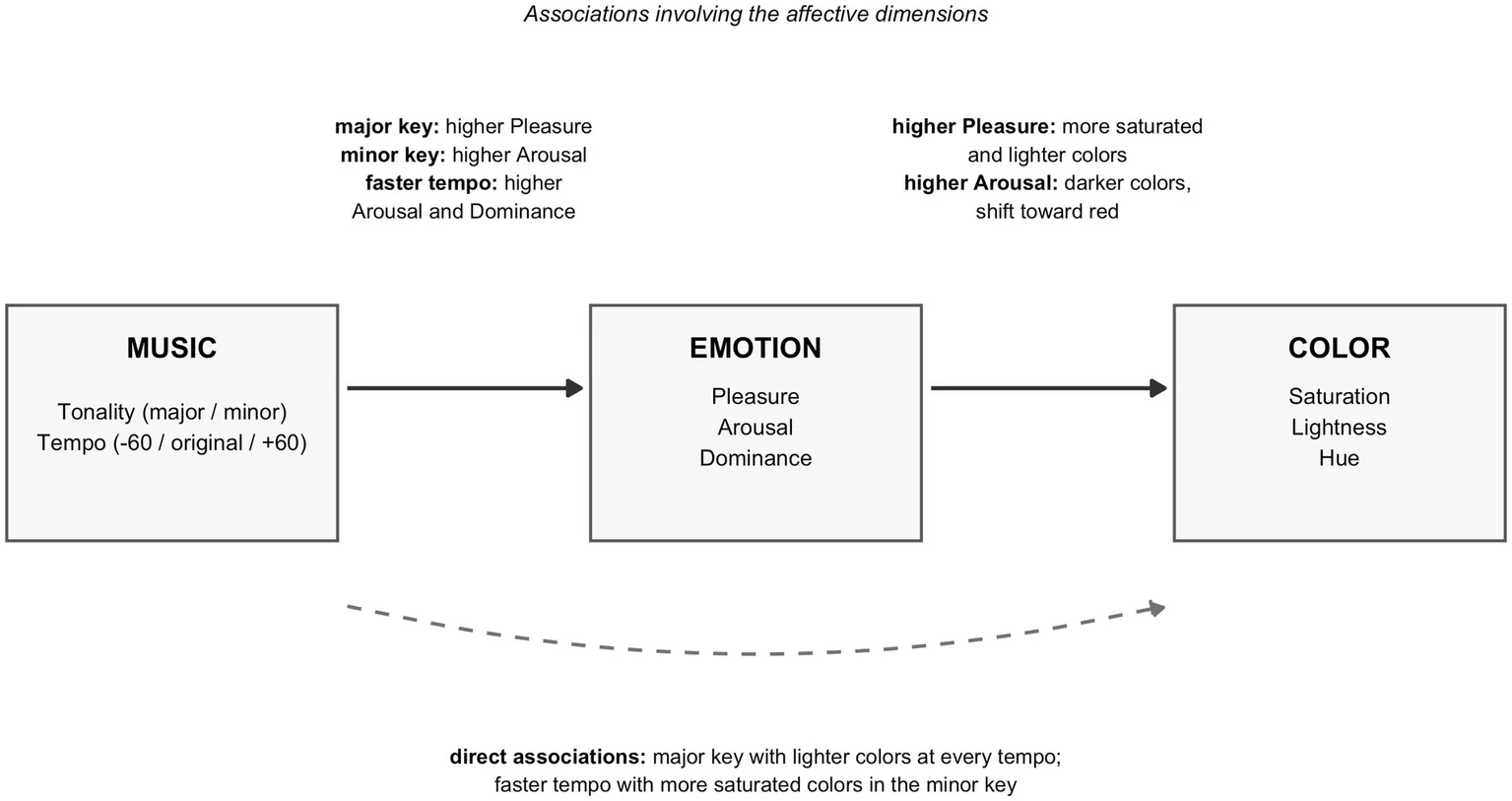
Schematic summary of the associations observed in the study: Solid arrows indicate the associations between musical parameters and affective dimensions, and between affective dimensions and color properties; the dashed arrow indicates the associations observed directly between musical parameters and color properties. The figure summarizes observed associations and does not represent a tested mediation model.

**Table 5:** Summary of the direction of the associations between PAD dimensions and chromatic properties.

PAD dimension	Saturation	Lightness	Hue (direction)	Red	Green	Blue
Pleasure	↑***	↑***	toward yellow-green***	↑***	↑***	↑***
Arousal	–	↓**	toward red***	–	–	↓***
Dominance	–	–	–	–	–	↓***

Arrows indicate the direction of the association estimated in the GEE models of Table 3 (↑ = increase; ↓ = decrease; — = non-significant). Hue directions derive from the circular (sine/cosine) components and indicate the resultant direction. *p < 0.05, **p < 0.01, ***p < 0.001.

## 4 Discussion

### 4.1 Overview of main findings

The present study investigated the relationship between music, emotion, and color by examining how musical parameters (tonality and tempo) relate to both emotional responses and color selections. Participants provided free-text emotional responses coded into the Pleasure-Arousal-Dominance (PAD) model (Russell and Mehrabian, 1974; Valdez and Mehrabian, 1994) and selected colors using an HSL-based picker, with responses analyzed using generalized estimating equations (GEE). The findings show that emotional dimensions systematically predict color selection, consistent with an emotion-mediated account of the associations between musical features and color, as captured by the HSL color space, a psychologically interpretable representation where Hue represents the chromatic family, Saturation the color intensity, and Lightness the perceived brightness. Altogether, the present findings describe a fluid and context-sensitive network of correspondences rather than a fixed or universal mapping between sound and color. The primary analyses focused on Hue, Saturation, and Lightness (Table 3), with RGB values retained as a complementary description of participants’ digital color output.

### 4.2 The role of emotion in music-color correspondences

Cross-modal correspondences between sound and color may arise through several mechanisms, including structural, statistical, and semantically mediated associations (Spence, 2011). The present data are consistent with the emotion-mediation account (Palmer et al., 2013; Lindborg and Friberg, 2015), according to which listeners do not match auditory and visual features directly but filter sound through emotional experience, and then map those emotional dimensions onto color properties. Lindborg and Friberg (2015) showed that color associations with music are mediated by emotion, and Malfatti et al. (2014) reported analogous evidence for shape-to-color associations in non-synesthetes. Our results extend this framework: Pleasure, Arousal, and Dominance scores derived from participants’ own emotional labels predicted Saturation, Lightness, and Hue choices, and these associations remained stable after controlling for tonality and tempo (Section 3.4.1), consistent with emotion acting as a bridge between modalities.

### 4.3 Lightness, pitch height, and pleasure

A robust finding in the cross-modal literature is that auditory dimensions map onto visual lightness: in Marks (1974) classical matching studies, nearly all participants set increasing pitch to increasing brightness, a pattern that, as Marks himself noted, mimics the correspondences reported in sound-color synesthesia despite being observed in non-synesthetes. More recently, Zelazny and Sørensen (2025), in a large-scale study (*N* = 6,626), found that pitch height was linearly mapped onto lightness, that this mapping was context-dependent (ascending sequences produced lighter choices than descending ones), and that lightness emerged as the primary factor for color choices, with saturation and hue showing secondary, context-modulated patterns. Our findings integrate naturally with this literature through the emotional route. Lightness increased with Pleasure (approximately +1.89 units per +0.10 in Pleasure) and decreased with Arousal (Table 3 and Figure 5). Moreover, minor-key excerpts were associated with lower Lightness at every tempo (Section 3.3.2), replicating the association between minor mode and darker color choices reported by Palmer et al. (2013), and the original-tempo condition was associated with slightly higher Lightness (*β* = +2.42, *p* = 0.022). Thus, both the direct musical effects and the affective effects on color converge on Lightness as a primary organizing dimension of the response, in line with Zelazny and Sørensen (2025).

### 4.4 Saturation and sensory intensity

Saturation showed a parallel pattern: it increased with Pleasure (approximately +1.80 units per +0.10 in Pleasure) and, in the minor key, with faster tempo (Table 3; Section 3.3.2). This is consistent with studies linking auditory intensity dimensions to chroma. Hamilton-Fletcher et al. (2017), using physically equiluminant colors, found that increases in auditory frequency and loudness were associated with increases in chroma. Ward et al. (2006) reported that both synesthetes and controls use similar heuristics when matching sounds of varying pitch and timbre to colors, indicating that these mappings rely on cross-modal mechanisms common to the general population rather than on mechanisms exclusive to synesthesia. In our data, the emotional counterpart of these effects, more pleasant, more energetic excerpts associated with more saturated choices, suggests that perceived intensity may operate at both the sensory and the affective level.

### 4.5 Hue: weaker and more context-dependent associations

Hue, modeled as a circular variable, showed weaker and more dispersed associations (global mean direction θ = 288°, *R* = 0.083), indicating high inter-individual variability in chromatic family choices (Figure 4). Nevertheless, the affective structure was detectable: Pleasure was associated with the sine component (shifts toward the yellow-green region) and Arousal with both components, including a shift toward the red region (Table 3). At the level of specific emotional labels, the associations were considerably sharper: words denoting anger clustered in the red-magenta region (mean direction ≈ 345°, *R* = 0.61) and words denoting sadness in the blue-cyan region (≈ 231°, *R* = 0.61) (Figure 6), consistent with the classical color-emotion literature (Valdez and Mehrabian, 1994; Elliot and Maier, 2014). Arousal ratings for calibrated Munsell hues presented in immersive virtual reality similarly peak for red and red-purple (Febbraio et al., 2025), converging with the arousal-red association observed here. The relative weakness of hue effects at the aggregate level, together with their specificity at the label level, fits the conclusion of Zelazny and Sørensen (2025) that hue associations are secondary and context-dependent, whereas lightness (and, in our data, saturation) carries the primary affective signal.

### 4.6 Affective responses to tonality and tempo

The affective results confirmed and refined the expected patterns (Tables 1, 2 and Figure 1). Pleasure peaked at the original tempo in the major key (slowed vs. original, *p* < 0.001; original vs. accelerated, *p* = 0.037), whereas in the minor key it increased from the slowed to the original tempo and then plateaued; excerpts in the major key were rated as more pleasant than minor-key excerpts at every tempo. Arousal was higher for minor-key excerpts at all tempi and increased with tempo in the minor key, with a reliable rise from the original to the accelerated condition but not from the slowed to the original one, while in the major key it did not increase beyond the original tempo. Dominance increased from the slowed to the original tempo in both tonalities but did not differ reliably between major and minor keys. These patterns are consistent with the literature on tempo and mode as determinants of musical emotion (Bigand et al., 2005; Hunter et al., 2010; Gagnon and Peretz, 2003). Linking the affective and chromatic results, the tempo-related increase in arousal for minor-key excerpts was accompanied by darker color choices (lower Lightness) and by hue shifts toward the red region, while pleasure tracked lighter and more saturated choices. The data thus describe a coherent affective gradient in color space: activation is associated with darker, warmer choices, and pleasantness with lighter, more vivid ones (Elliot and Maier, 2014; Valdez and Mehrabian, 1994). The absolute-BPM control analysis confirmed this reading: Pleasure, Arousal, Saturation and Lightness all increased slightly with faster absolute tempo, and the affective effects on color remained unchanged when BPM was controlled for (Section 3.6).

### 4.7 Variability and cultural mediation of color-emotion mappings

The high inter-individual variability observed for hue has already been documented in the literature, both for color selection and for the corresponding emotional associations, and in the broader body of research suggesting that the meaning attributed to colors can vary significantly depending on cultural background (Shaver et al., 1987; Elliot and Maier, 2014; Valdez and Mehrabian, 1994; Jonauskaite et al., 2024; Jonauskaite and Mohr, 2025). Recent large-scale cross-cultural analyses confirm this variability (Jonauskaite et al., 2024; Jonauskaite and Mohr, 2025): sociocultural learning and exposure can influence the emotional valence of colors, particularly red and blue (Elliot and Maier, 2014; Jonauskaite et al., 2024). It is likely that personal mental imagery and learned associations also play a role, where listeners do not respond to sound in a standardized way but filter it through memories and expectations that can affect both chromatic and emotional responses. Similarly, findings regarding the influence of music on emotional responses and color associations reveal a comparable complexity to that found in studies examining color-emotion pairings alone. However, the relationship between warm colors (e.g., red) and positive emotions (e.g., joy) and between cold colors (e.g., blue) and negatively valenced emotions (e.g., melancholy) appears to be far more nuanced. These associations vary with musical parameters such as key and tempo, indicating a more intricate interplay among perceptual and emotional dimensions (Carraturo et al., 2025; Gagnon and Peretz, 2003; Hunter et al., 2010; Wilms and Oberfeld, 2018; Palmer et al., 2013). This supports the idea that listeners may form flexible correspondences between sound and color, relating emotional qualities rather than matching sensory channels directly (Carraturo et al., 2025; Wilms and Oberfeld, 2018; Palmer et al., 2013).

### 4.8 Limitations and future directions

Several limitations must be acknowledged. First, beyond the exclusion of participants with formal musical training or regular classical-music listening, no further individual background information (age, gender, education, cultural background, or general artistic experience) was systematically collected; the findings therefore apply to the specific sample tested (predominantly Italian university students) and cannot be generalized to the broader population. Second, tonality and pitch class were confounded with musical excerpt: major and minor conditions were based on different pieces, and each pitch class was represented by a single excerpt. All tonality- and note-related findings should therefore be read as associations with specific excerpts rather than as effects of tonality or pitch class per se; disentangling these factors would require transformed-melody designs or multiple excerpts per condition with item-level variability modeled statistically. Third, tempo categories were defined relative to each excerpt’s original tempo, so absolute BPM values varied widely within categories, with within-category variability exceeding the between-category gap, although the categories themselves did not overlap; the control analysis with absolute BPM as a continuous covariate confirmed the reported effects, but future designs should standardize absolute tempo ranges. Fourth, no instrumental colorimetric calibration of the display was performed, and color-vision deficiencies were excluded only by self-report, which cannot rule out undiagnosed cases. Fifth, because each design cell contained a single excerpt, item-level random effects could not be estimated. Finally, participants were not screened for synesthesia or synesthetic tendencies, and the present behavioral data cannot speak to the neural mechanisms underlying the observed associations; in particular, because “pitch class” here denotes the key of each excerpt rather than the chroma of individual tones, our data cannot address tone-level pitch class-color associations such as those documented in pitch class-color synesthesia among possessors of absolute pitch (Itoh et al., 2019). Future research should employ demographically characterized and cross-cultural samples, controlled stimulus transformations, calibrated displays, and multiple excerpts per condition, and could complement the dimensional PAD approach with discrete emotion frameworks (Zentner et al., 2008).

### 4.9 Conclusion

In conclusion, the interrelations between emotional, perceptual, and creative dimensions of human experience have long been explored by artists, psychologists, and neuroscientists. The present study shows that the emotional dimensions expressed by listeners in their own words systematically predict the colors they choose: pleasure was associated with lighter and more saturated choices, arousal with darker and warmer ones, and these associations remained stable across tonality, tempo, and analytic specifications. These findings are consistent with an emotion-mediated account of music-color correspondences (Spence, 2011; Palmer et al., 2013; Lindborg and Friberg, 2015) and converge with evidence that lightness is the primary dimension of cross-modal color choice (Marks, 1974; Zelazny and Sørensen, 2025). Despite the substantial inter-individual variability documented in color-emotion research (Jonauskaite et al., 2024; Jonauskaite and Mohr, 2025), the affective organization of color choices was nonetheless systematic at the group level. By bridging perceptual, affective, and aesthetic domains, this research contributes to a growing body of evidence on how affective experience relates to the associations people form between sound and color.

## Data Availability

The raw data supporting the conclusions of this article will be made available by the authors, without undue reservation.
