# Supplementary material for: Colored tones of emotions: the relationship between music, emotions and color
Source: Front Psychol. 2026 Sep 16;17:1777421. doi: 10.3389/fpsyg.2026.1777421 (PMC13623571; doi:10.3389/fpsyg.2026.1777421)
Supplement: Supplementary file 1 [file Supplementary_file_1.DOCX]

Supplementary Material

Colored Tones of Emotions: The Relationship between Music, Emotions and Color

Febbraio F., De Simone F., Taiani C., Collina S.

This Supplementary Material provides design and coding materials that support the reproducibility of the study but are not reproduced in full in the main text: the complete list of musical stimuli (Supplementary Table S1), the mapping of the emotion labels onto NRC-VAD scores (Supplementary Table S2), the verbatim participant instructions (Supplementary Appendix S1), and the codebook used for the manual PAD coding and for the independent second rater (Supplementary Appendix S2). All statistical results are reported in full in the main text.

# Supplementary Table S1

**Table S1. Musical stimuli employed in the experiment.**

List of selected excerpts organized by pitch class (C–B) and tonality (major/minor). For each piece, the original tempo (BPM), manipulated tempi (±60 BPM), and excerpt start time are reported. An additional filler condition was included using John Cage’s Living Room Music as an aleatoric contrast. The final three rows list the non-experimental excerpts used for the three practice trials described in Section 2.4; these were presented at their original tempo only and were excluded from all analyses.

| **Pitch Class** | **Tonality** | **Piece (Abbreviated Title)** | **Original BPM** | **+60** | **–60** | **Start time (mm:ss)** |
| --- | --- | --- | --- | --- | --- | --- |
| C | C Minor | Chopin – Nocturne No.20 | 114 | 174 | 54 | 0:24 |
| C | C Major | Bach – Preludio | 120 | 180 | 60 | 0:00 |
| D | D Minor | D. Scarlatti – Sonata K1 | 100 | 160 | 40 | 0:00 |
| D | D Major | N. Porpora – Sinfonia da Camera | 120 | 180 | 60 | 1:33 |
| E | E Minor | Mozart – Sonata K.304 | 97 | 157 | 37 | 0:45 |
| E | E Major | Piccinni – Sonata in E Major | 91 | 151 | 31 | 0:00 |
| F | F Minor | Schubert – Fantasia in F Minor | 120 | 180 | 60 | 14:35 |
| F | F Major | Cimarosa – Sestetto | 120 | 180 | 60 | 13:10 |
| G | G Minor | Pergolesi – Nina | 84 | 144 | 24 | 0:00 |
| G | G Major | Bach – Cello Suite No.1 | 86 | 146 | 26 | 0:00 |
| A | A Minor | Mahler – Piano Quartet | 105 | 165 | 45 | 4:40 |
| A | A Major | Durante – Concerto a cinque | 110 | 170 | 50 | 2:39 |
| B | B Minor | Pergolesi – Alla Siciliana | 83 | 143 | 23 | 4:45 |
| B | B Major | D. Scarlatti – Sonata K266 | 83 | 143 | 23 | 0:40 |
| — | Aleatoric | John Cage – *Living Room Music* | — | *Filler* | | randomized |
| D | Major | P. Watson - Je te laisserai des mots | 133 | *Training* | | 0:00 |
| F | Minor | Coldplay - Viva la Vida | 138 | *Training* | | 0:00 |
| D | Minor | Jane Birkin - Di doo dah | 76 | *Training* | | 0:00 |

*Note. All excerpts were standardized to 30 seconds in duration across tempo conditions. For slowed versions (−60 BPM), segments were truncated at 30 seconds; for accelerated versions (+60 BPM), longer source passages were extracted so that the final excerpts also lasted 30 seconds. This procedure ensured constant trial duration across all conditions while maintaining the integrity of tempo manipulations.*

**Supplementary Table S2**

**Italian-to-English mapping of the emotion labels and corresponding NRC-VAD scores.**

Complete list of the 164 distinct Italian emotion labels produced by participants, with their frequency of occurrence across the 1,680 trials, the English term to which each was mapped, and the corresponding Pleasure, Arousal and Dominance values. Normative Pleasure, Arousal and Dominance values are taken from the NRC Valence-Arousal-Dominance Lexicon v2.1 (Mohammad, 2018, 2025), used for non-commercial research in accordance with its terms of use. Source: http://saifmohammad.com/WebPages/nrc-vad.html

| **Italian label** | **n** | **English translation** | **Pleasure** | **Arousal** | **Dominance** |
| --- | --- | --- | --- | --- | --- |
| abbandono | 4 | abandonment | 0.128 | 0.430 | 0.202 |
| affascinata | 2 | fascinated | 0.812 | 0.714 | 0.788 |
| agitazione | 34 | agitation | 0.240 | 0.931 | 0.447 |
| agonia | 2 | agony | 0.083 | 0.775 | 0.393 |
| allegria | 61 | cheerfulness | 0.969 | 0.802 | 0.675 |
| altruismo | 1 | altruism | 1.000 | 0.572 | 0.604 |
| amarezza | 9 | bitterness | 0.115 | 0.633 | 0.270 |
| ambiguità | 1 | ambiguity | 0.164 | 0.460 | 0.370 |
| ammirazione | 4 | admiration | 0.969 | 0.583 | 0.726 |
| amore | 15 | love | 0.998 | 0.667 | 0.617 |
| angoscia | 29 | distress | 0.073 | 0.840 | 0.396 |
| ansia | 88 | anxiety | 0.146 | 0.865 | 0.342 |
| appagamento | 4 | contentment | 0.875 | 0.610 | 0.782 |
| armonia | 4 | harmony | 0.949 | 0.230 | 0.509 |
| attenzione | 1 | attention | 0.740 | 0.599 | 0.774 |
| attesa | 3 | anticipation | 0.698 | 0.539 | 0.711 |
| benessere | 4 | wellbeing | 1.000 | 0.000 | 0.389 |
| calma | 132 | calm | 0.875 | 0.050 | 0.314 |
| certezza | 1 | certainty | 0.781 | 0.400 | 0.769 |
| commozione | 6 | moved | 0.762 | 0.760 | 0.416 |
| complicità | 2 | closeness | 0.796 | 0.237 | 0.450 |
| concentrazione | 5 | concentration | 0.735 | 0.500 | 0.722 |
| condanna | 1 | condemnation | 0.133 | 0.725 | 0.449 |
| confusione | 16 | confusion | 0.255 | 0.667 | 0.277 |
| convivialità | 2 | conviviality | 1.000 | 0.834 | 0.796 |
| cullante | 2 | soothing | 0.625 | 0.179 | 0.519 |
| curiosità | 5 | curiosity | 0.750 | 0.755 | 0.463 |
| delicatezza | 1 | gentleness | 0.927 | 0.278 | 0.447 |
| delusione | 11 | disappointment | 0.115 | 0.490 | 0.336 |
| depressione | 3 | depression | 0.031 | 0.520 | 0.232 |
| determinazione | 8 | determination | 0.776 | 0.725 | 0.795 |
| difficoltà | 1 | difficulty | 0.123 | 0.675 | 0.279 |
| disagio | 5 | discomfort | 0.153 | 0.719 | 0.272 |
| disgusto | 4 | disgust | 0.052 | 0.775 | 0.317 |
| disperazione | 20 | despair | 0.115 | 0.794 | 0.245 |
| disturbo | 1 | disturbance | 0.146 | 0.907 | 0.404 |
| divertimento | 8 | fun | 0.918 | 0.843 | 0.667 |
| dolcezza | 6 | sweetness | 0.906 | 0.474 | 0.528 |
| dolore | 10 | pain | 0.041 | 0.765 | 0.325 |
| drammaticità | 3 | intensity | 0.622 | 0.930 | 0.952 |
| eleganza | 3 | elegance | 0.918 | 0.396 | 0.824 |
| elettricità | 2 | excitement | 0.896 | 0.684 | 0.731 |
| emozionata | 2 | excited | 0.908 | 0.931 | 0.709 |
| emozionato | 1 | excited | 0.908 | 0.931 | 0.709 |
| energia | 7 | energy | 0.944 | 0.725 | 0.868 |
| energica | 4 | energetic | 0.847 | 0.868 | 0.902 |
| entusiasmo | 17 | enthusiasm | 0.819 | 0.757 | 0.798 |
| epico | 1 | epic | 0.857 | 0.713 | 0.830 |
| equilibrio | 1 | balance | 0.684 | 0.265 | 0.735 |
| estasi | 2 | ecstasy | 0.796 | 0.922 | 0.655 |
| euforia | 13 | euphoria | 0.769 | 0.955 | 0.648 |
| fallimento | 1 | failure | 0.167 | 0.575 | 0.139 |
| falsità | 1 | deception | 0.090 | 0.745 | 0.370 |
| fastidio | 9 | annoyance | 0.167 | 0.718 | 0.342 |
| felicità | 75 | happiness | 0.960 | 0.732 | 0.850 |
| forza | 3 | strength | 0.802 | 0.682 | 0.858 |
| forzatura | 4 | coercion | 0.330 | 0.708 | 0.577 |
| fretta | 6 | hurry | 0.422 | 0.735 | 0.481 |
| frettolosità | 1 | haste | 0.378 | 0.745 | 0.500 |
| frustrazione | 5 | frustration | 0.060 | 0.730 | 0.280 |
| gioia | 85 | joy | 0.980 | 0.824 | 0.794 |
| grandiosità | 3 | grandiosity | 0.666 | 0.834 | 0.979 |
| imbarazzo | 2 | embarrassment | 0.143 | 0.685 | 0.226 |
| imbroglio | 2 | deception | 0.090 | 0.745 | 0.370 |
| impazienza | 3 | impatience | 0.278 | 0.780 | 0.411 |
| impetuosità | 1 | impulsiveness | 0.166 | 1.000 | 0.233 |
| imponenza | 1 | majesty | 0.823 | 0.602 | 0.978 |
| impulsività | 2 | impulsivity | 0.195 | 0.834 | 0.209 |
| incerto | 1 | uncertain | 0.363 | 0.500 | 0.164 |
| incoraggiante | 2 | encouraging | 0.823 | 0.583 | 0.755 |
| indaffarato | 1 | busy | 0.402 | 0.500 | 0.474 |
| indecisione | 1 | indecision | 0.184 | 0.480 | 0.196 |
| indignazione | 1 | indignation | 0.173 | 0.778 | 0.431 |
| inebriata | 1 | exhilarated | 0.890 | 0.982 | 0.671 |
| infatuazione | 2 | infatuation | 0.844 | 0.745 | 0.530 |
| inferiorità | 1 | inferiority | 0.177 | 0.430 | 0.214 |
| inquietudine | 14 | restlessness | 0.083 | 0.794 | 0.397 |
| insoddisfazione | 1 | dissatisfaction | 0.115 | 0.663 | 0.202 |
| instabilità | 2 | instability | 0.177 | 0.760 | 0.353 |
| interesse | 1 | interest | 0.780 | 0.667 | 0.618 |
| intraprendenza | 1 | initiative | 0.833 | 0.615 | 0.768 |
| introspettività | 1 | introspection | 0.603 | 0.337 | 0.575 |
| inutile | 2 | useless | 0.052 | 0.342 | 0.195 |
| irrequietezza | 6 | restlessness | 0.083 | 0.794 | 0.397 |
| leggerezza | 17 | lightness | 0.794 | 0.360 | 0.526 |
| libertà | 11 | freedom | 0.969 | 0.544 | 0.905 |
| magnificenza | 1 | magnificence | 0.802 | 0.810 | 0.920 |
| malessere | 2 | distress | 0.073 | 0.840 | 0.396 |
| malinconia | 93 | melancholy | 0.188 | 0.260 | 0.237 |
| maliziosità | 2 | mischief | 0.385 | 0.834 | 0.292 |
| malvagità | 2 | wickedness | 0.031 | 0.727 | 0.443 |
| meraviglia | 1 | wonder | 0.939 | 0.771 | 0.765 |
| motivato | 2 | motivated | 0.834 | 0.854 | 0.979 |
| neutro | 1 | neutral | 0.469 | 0.184 | 0.357 |
| noia | 44 | boredom | 0.167 | 0.139 | 0.255 |
| nostalgia | 38 | nostalgia | 0.417 | 0.337 | 0.339 |
| orgoglio | 1 | pride | 0.729 | 0.634 | 0.848 |
| orrore | 2 | horror | 0.115 | 0.850 | 0.438 |
| ossessione | 1 | obsession | 0.229 | 0.847 | 0.571 |
| pace | 16 | peace | 0.970 | 0.078 | 0.613 |
| panico | 2 | panic | 0.062 | 0.938 | 0.400 |
| passione | 7 | passion | 0.854 | 0.865 | 0.728 |
| paura | 31 | fear | 0.073 | 0.840 | 0.293 |
| pazzia | 2 | madness | 0.104 | 0.804 | 0.440 |
| pensieroso | 3 | thoughtful | 0.667 | 0.290 | 0.527 |
| pesantezza | 1 | heaviness | 0.219 | 0.549 | 0.318 |
| piacere | 5 | pleasure | 0.939 | 0.740 | 0.682 |
| piccolezza | 1 | insignificance | 0.250 | 0.216 | 0.222 |
| pietà | 1 | pity | 0.160 | 0.398 | 0.114 |
| potenza | 2 | power | 0.719 | 0.670 | 0.976 |
| pressione | 4 | pressure | 0.360 | 0.650 | 0.623 |
| profondità | 2 | depth | 0.708 | 0.561 | 0.759 |
| rabbia | 56 | anger | 0.167 | 0.865 | 0.657 |
| rassegnazione | 17 | resignation | 0.145 | 0.392 | 0.310 |
| realizzazione | 2 | accomplishment | 0.917 | 0.694 | 0.888 |
| relax | 33 | relaxation | 0.873 | 0.238 | 0.381 |
| riflessione | 2 | reflection | 0.710 | 0.500 | 0.528 |
| rimorso | 2 | remorse | 0.103 | 0.673 | 0.377 |
| rimpianto | 6 | regret | 0.230 | 0.623 | 0.331 |
| rivalsa | 1 | revenge | 0.230 | 0.940 | 0.627 |
| romanticismo | 3 | romance | 0.980 | 0.682 | 0.587 |
| sacrificio | 1 | sacrifice | 0.208 | 0.833 | 0.534 |
| sconforto | 2 | discouragement | 0.112 | 0.319 | 0.206 |
| sentimentale | 2 | sentimental | 0.583 | 0.378 | 0.312 |
| serenità | 58 | serenity | 0.900 | 0.061 | 0.557 |
| serietà | 2 | seriousness | 0.439 | 0.365 | 0.750 |
| sgomento | 1 | dismay | 0.314 | 0.681 | 0.459 |
| sicurezza | 5 | safety | 0.708 | 0.491 | 0.868 |
| sicuro | 2 | safe | 0.898 | 0.306 | 0.759 |
| sinuosità | 1 | gracefulness | 1.000 | 0.334 | 0.574 |
| sobrietà | 1 | restraint | 0.167 | 0.618 | 0.520 |
| soddisfazione | 4 | satisfaction | 0.939 | 0.637 | 0.673 |
| sofferenza | 5 | suffering | 0.020 | 0.719 | 0.235 |
| sognatore | 5 | dreamer | 0.820 | 0.394 | 0.455 |
| solennità | 1 | solemnity | 0.334 | 0.062 | 0.785 |
| solitudine | 15 | loneliness | 0.146 | 0.182 | 0.222 |
| sollievo | 14 | relief | 0.844 | 0.278 | 0.481 |
| sorpresa | 5 | surprise | 0.875 | 0.875 | 0.562 |
| sottomissione | 1 | submission | 0.531 | 0.580 | 0.633 |
| spensieratezza | 32 | carefree | 0.755 | 0.220 | 0.500 |
| speranza | 6 | hope | 0.927 | 0.317 | 0.739 |
| stanchezza | 2 | tiredness | 0.387 | 0.408 | 0.242 |
| stranezza | 1 | weirdness | 0.316 | 0.491 | 0.364 |
| strazio | 2 | anguish | 0.153 | 0.792 | 0.265 |
| stress | 7 | stress | 0.173 | 0.843 | 0.317 |
| struggente | 1 | poignant | 0.646 | 0.444 | 0.518 |
| stupita | 2 | surprised | 0.784 | 0.855 | 0.539 |
| stupore | 1 | amazement | 0.750 | 0.800 | 0.639 |
| suspance | 4 | suspense | 0.286 | 0.775 | 0.402 |
| tenacia | 1 | tenacity | 0.740 | 0.657 | 0.889 |
| tensione | 26 | tension | 0.304 | 0.810 | 0.529 |
| terrore | 4 | terror | 0.069 | 0.902 | 0.415 |
| tormento | 3 | torment | 0.057 | 0.750 | 0.387 |
| tranquillità | 63 | tranquility | 0.840 | 0.135 | 0.442 |
| trasporto | 2 | passion | 0.854 | 0.865 | 0.728 |
| travolgente | 1 | overwhelming | 0.327 | 0.830 | 0.640 |
| trionfo | 1 | triumph | 0.969 | 0.798 | 0.926 |
| tristezza | 151 | sadness | 0.052 | 0.288 | 0.164 |
| vigile | 1 | alertness | 0.375 | 0.875 | 0.750 |
| vitalità | 1 | vitality | 0.923 | 0.565 | 0.869 |
| vittoria | 2 | victory | 0.938 | 0.735 | 0.931 |
| vivacità | 7 | liveliness | 1.000 | 1.000 | 0.750 |
| voglia | 1 | desire | 0.896 | 0.692 | 0.647 |
| vuoto | 2 | emptiness | 0.177 | 0.173 | 0.184 |

*Note. Free-text responses were normalized (lowercase, accent and whitespace normalization, lemmatization) and translated into English using the Oxford English Dictionary, selecting the sense corresponding to an emotional state for polysemous terms. The 164 distinct Italian labels mapped onto 159 distinct English entries: one pair reflected grammatical gender inflection of the same adjective (emozionata / emozionato → excited), and four pairs were near-synonyms sharing a single English equivalent (angoscia / malessere → distress; falsità / imbroglio → deception; inquietudine / irrequietezza → restlessness; passione / trasporto → passion). No labels were merged beyond these cases, and no response was excluded. Pleasure, Arousal and Dominance values are normative lexicon values for the selected English term (NRC-VAD v2.1, rescaled to 0–1): they describe the affective properties conventionally associated with that word and are* ***not*** *intensity ratings provided by participants.*

# Supplementary Appendix S1. Participant Instructions (verbatim)

Instructions were administered in Italian; an English translation is provided below. Task order (emotion first vs. color first) was counterbalanced across participants; the two versions of the instructions were identical except for the order of the two tasks.

**Condition 1: Emotion → Color**

"In this experiment you will listen to a series of musical excerpts. The music will start automatically. While listening, you can provide your responses at any time, without waiting for the end of the excerpt. You will be asked to perform two tasks: (1) Write the name of an emotion: freely enter the name of the emotion you are feeling while listening to the excerpt. (2) Select a color: to choose the color, click on the box showing the color code (e.g., #000000). A color picker will open: use the mouse to choose the color you consider most representative of your experience. Click "OK" to confirm your selection. After completing both responses, click "Next" to move to the following excerpt. Please note: there are no right or wrong answers; you may use the same emotion or the same color for different excerpts; you may respond at any moment during listening, following your immediate or more reflective experience."

**Condition 2: Color → Emotion**

Identical to Condition 1, with the two tasks presented in reverse order (color selection first, emotion label second).

### Supplementary Appendix S2. Codebook for the Manual PAD Coding

The following coding scheme (translated from Italian) was used for the manual dichotomous coding of the emotion labels, and was given verbatim to the independent second coder. The task consisted of classifying each of the 164 distinct Italian emotion words, indicating for each word whether it expresses each of the three affective dimensions of the Pleasure-Arousal-Dominance model (1 = dimension present; 0 = dimension absent). Coders were instructed to: judge each word in its common meaning, as an emotional state experienced by a person; treat the three dimensions as independent; choose the predominant pole for ambiguous or polysemous words; work alone, without consulting colleagues, emotion dictionaries, or study materials; and complete the coding in one or two sessions without reordering the (randomized) word list.

| **Dimension** | **Code 1 (present)** | **Code 0 (absent)** |
| --- | --- | --- |
| **Pleasure** (pleasantness/valence) | The word describes a state that is pleasant, positive, agreeable for the person experiencing it (well-being, satisfaction, joy...). | The word describes an unpleasant, negative, or neutral-negative state (discomfort, suffering, malaise...). |
| **Arousal** (activation) | The state involves activation, energy, excitement, agitation—body and mind "switched on" (racing heart, tension, alertness...). | The state is calm, relaxed, deactivated, low-energy (quiet, torpor, relaxation...). |
| **Dominance** (perceived control/power) | A person experiencing this state feels in control of the situation, strong, self-possessed. | A person experiencing this state feels controlled by events, powerless, overwhelmed, at the mercy of the situation. |

*Note. Two anchor examples from the PAD literature (Mehrabian & Russell, 1974; Russell & Mehrabian, 1977) were provided for calibration: fear (P = 0, A = 1, D = 0) and calm (P = 1, A = 0, D = 1). All other words were judged autonomously. Inter-rater reliability statistics (Cohen's kappa) and convergent-validity analyses with the NRC-VAD lexicon are reported in full in the main text (Sections 2.6.2).*
